# Caspase-8 mutations associated with head and neck cancer differentially retain functional properties related to TRAIL-induced apoptosis and cytokine induction

**DOI:** 10.1038/s41419-021-04066-z

**Published:** 2021-08-06

**Authors:** Zhibin Cui, Hadas Dabas, Brandon C. Leonard, Jamie V. Shiah, Jennifer R. Grandis, Daniel E. Johnson

**Affiliations:** Department of Otolaryngology—Head and Neck Surgery, University of California at San Francisco, San Francisco, CA USA

**Keywords:** Medical research, Preclinical research

## Abstract

The cysteine protease, caspase-8, undergoes dimerization, processing, and activation following stimulation of cells with death ligands such as TRAIL, and mediates TRAIL induction of the extrinsic apoptosis pathway. In addition, caspase-8 mediates TRAIL-induced activation of NF-κB and upregulation of immunosuppressive chemokines/cytokines, via a mechanism independent of caspase-8 catalytic activity. The gene encoding procaspase-8 is mutated in 10% of human head and neck squamous cell carcinomas (HNSCCs). Despite a paucity of experimental evidence, HNSCC-associated caspase-8 mutations are commonly assumed to be loss of function. To investigate their functional properties and phenotypic effects, 18 HNSCC-associated caspase-8 mutants were expressed in doxycycline-inducible fashion in cell line models wherein the endogenous wild-type caspase-8 was deleted. We observed that 5/8 mutants in the amino-terminal prodomain, but 0/10 mutants in the carboxyl-terminal catalytic region, retained an ability to mediate TRAIL-induced apoptosis. Caspase-8 proteins with mutations in the prodomain were defective in dimerization, whereas all ten of the catalytic region mutants efficiently dimerized, revealing an inverse relationship between dimerization and apoptosis induction for the mutant proteins. Roughly half (3/8) of the prodomain mutants and 9/10 of the catalytic region mutants retained the ability to mediate TRAIL induction of immunosuppressive CXCL1, IL-6, or IL-8. Doxycycline-induced expression of wild-type caspase-8 or a representative mutant led to an increased percentage of T and NKT cells in syngeneic HNSCC xenograft tumors. These findings demonstrate that HNSCC-associated caspase-8 mutants retain properties that may influence TRAIL-mediated apoptosis and cytokine induction, as well as the composition of the tumor microenvironment.

## Introduction

Caspase-8 is an intracellular cysteine protease that is synthesized in a zymogen form and becomes processed and activated following ligand engagement of cell surface death receptors in the tumor necrosis factor receptor (TNFR) superfamily. Activated caspase-8 plays key roles in mediating induction of the extrinsic apoptosis pathway and suppression of necroptotic cell death [1–3]. In addition, caspase-8 acts via a mechanism independent of its catalytic activity to mediate death-ligand activation of NF-κB and upregulation of NF-κB target genes, including genes encoding inflammatory and immunosuppressive cytokines [4, 5]. Mutations in caspase-8 have been observed, albeit rarely, in human diseases resembling autoimmune lymphoproliferative syndrome where they contribute to immunodeficiency [6–8]. A large number of caspase-8 mutations have also been identified in human cancers, although the functional properties of these mutations and their role in cancer are poorly understood.

During activation of the extrinsic apoptosis pathway, the binding of death ligands such as TRAIL to their cognate receptors results in receptor recruitment of the death domain-containing adaptor protein FADD, initiating the formation of the death-inducing signaling complex. The carboxyl-terminal region of FADD contains a death effector domain (DED), which recruits procaspase-8 via a DED in the amino-terminal prodomain of the zymogen [3, 9, 10]. Dimerization or oligomerization of the recruited zymogen with another procaspase-8 molecule occurs via a recently elucidated domain-swap mechanism and results in autoprocessing/activation [11–14]. Activated caspase-8 cleaves and activates downstream executioner caspases, including caspase-3 and -7, which promote cellular destruction.

Caspase-8 also plays a key role in mediating TRAIL activation of NF-κB, via a mechanism that is independent of caspase-8 catalytic activity. In this pathway, stimulation with TRAIL leads to the formation of the “FADDosome”, a cytoplasmic complex consisting of FADD, procaspase-8, and RIPK1 [4, 15]. Procaspase-8 serves a scaffolding function in formation of the FADDosome. Formation of this complex leads to ubiquitination of RIPK1, which facilitates recruitment of the IKK and TAK1/TAB1/TAB2 complexes, resulting in phosphorylation and degradation of IκB and activation of NF-κB [3, 4]. Active NF-κB mediates the transcription of a large number of pro-survival genes, as well as genes encoding inflammatory and immunosuppressive chemokines and cytokines.

Genomic sequencing efforts by The Cancer Genome Atlas (TCGA) have revealed that caspase-8 is mutated in a number of solid tumors [16–18]. In particular, mutation of caspase-8 occurs in 10% of head and neck squamous cell carcinoma (HNSCC) tumors [19–22], a malignancy associated with tobacco and alcohol consumption and oropharyngeal infection with human papillomavirus [23]. Mutations of the *caspase-8* gene in HNSCC include both missense and nonsense mutations, are widely distributed throughout the protein-coding region, and are associated with reduced patient survival [19, 24]. While much has been learned about the diverse functions of wild-type caspase-8, primarily via gene knockout studies, the functional properties and phenotypic effects of cancer-associated caspase-8 mutations are largely unknown.

In this study, we sought to determine the impact of 18 HNSCC-associated caspase-8 mutations on TRAIL induction of caspase-8 dimerization, apoptosis, and chemokine/cytokine upregulation. The impact of a representative caspase-8 mutant on the tumor microenvironment in a syngeneic, immunocompetent mouse model of HNSCC was also investigated. We report that mutations in the amino-terminal prodomain largely abrogated TRAIL-induced dimerization, although roughly half of these mutants retained a weak capacity to mediate TRAIL induction of cell death. By contrast, while all mutants in the carboxyl-terminal catalytic domain retained dimerization capacity, none retained the capacity to mediate cell death. All but one of the carboxyl-terminal mutants, and approximately half of the amino-terminal mutants, mediated TRAIL upregulation of immunosuppressive IL-6, IL-8, or CXCL1, supporting a key tenet of the FADDosome model that the catalytic function of caspase-8 is not required. Lastly, the expression of wild-type caspase-8 or a representative caspase-8 mutant increased the percentage of T and NKT cells in syngeneic HNSCC xenograft tumors grown in wild-type mice. Collectively, our findings demonstrate that multiple, HNSCC-associated, caspase-8 mutants retain diverse functional properties and phenotypic effects and are not simple loss of functional proteins. Of particular importance, the mutant caspase-8 proteins may retain the capacity to mediate the death-ligand upregulation of immunosuppressive chemokines/cytokines and modulate the immune composition of HNSCC tumors.

## Results

### Exogenous expression of WT caspase-8, but not HNSCC-associated caspase-8 MTs, induces apoptosis

Our studies were aimed at determining the functional properties of 18 known HNSCC-associated caspase-8 mutants (MTs) reported by TCGA (279 HNSCC tumors; [19]) and a prior cohort of 74 HNSCC tumors [20]. We focused on mutants generated by nonsense and missense mutations. We were unable to study the impact of three short truncation mutants (R68*, E89*, Q97*) as their expression was undetectable in transfected cell lines. Among the 18 caspase-8 mutations studied, 4 occur at sites (G11, S375, S386, Q465) reported as being mutated in more than one HNSCC patient [19, 20].

**Fig. 1:**
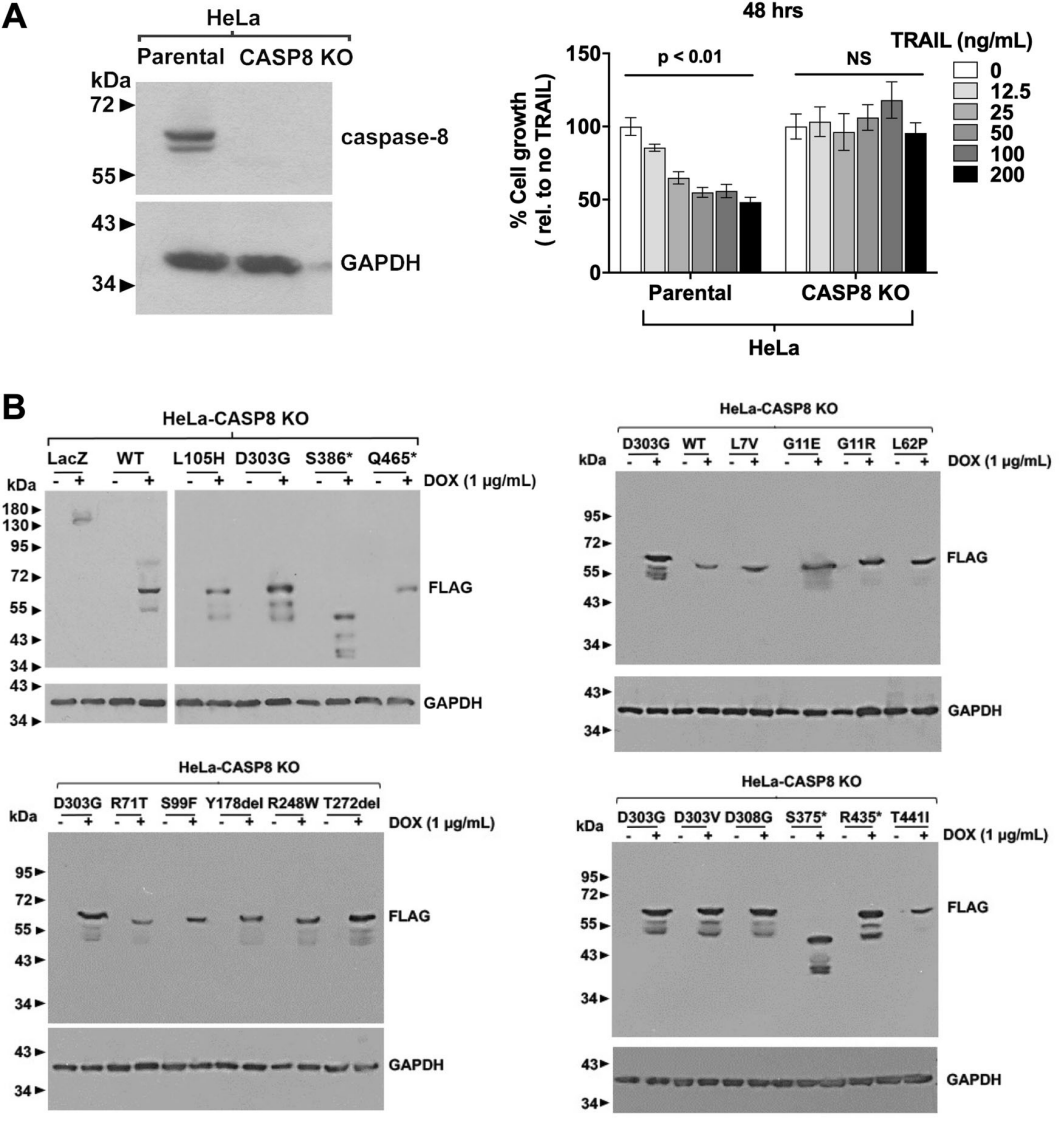
Exogenous expression of WT or MT caspase-8 in HeLa-CASP8 KO cells. **A** HeLa parental cells and HeLa-CASP8 KO cells were subjected to immunoblotting for caspase-8 or GAPDH (left panel). In the right panel, parental HeLa cells and HeLa-CASP8 KO cells were treated for 48 h with the indicated concentrations of TRAIL, followed by the performance of MTT assays. Data were plotted as percent of cell growth relative to no TRAIL treatment. Columns represent the means from five wells; error bars represent the SEM. One-way ANOVA was performed for the comparison of differences between multiple groups. The experiment was performed three times, with similar results. **B** HeLa-CASP8 KO cells engineered for DOX-inducible expression of FLAG-tagged, WT caspase-8, MT caspase-8 proteins, or the control protein LacZ, were treated for 24 h in the absence or presence of DOX (1 μg/mL), followed by immunoblotting with anti-FLAG. Immunoblotting for GAPDH was used as a loading control. To allow comparison of expression levels between gels, an equivalent amount of the D303G mutant was loaded on each gel. T272del indicates the T272_T273del mutant, an in-frame mutant lacking three nucleotides. The experiments were performed three times with similar results.

We selected HeLa cells as our primary model for exogenous expression and functional characterization of the caspase-8 mutants, as these cells activate cell death signaling and cytokine induction in a caspase-8-dependent manner following TRAIL treatment. To allow us to focus exclusively on the function of the mutant proteins in the absence of WT caspase-8, we first used CRISPR-based technology to delete the expression of endogenous WT caspase-8. The resulting HeLa-CASP8 KO cells demonstrated no expression of caspase-8 protein and, in contrast to parental HeLa cells, did not exhibit inhibition of cell growth (Fig. 1A) following TRAIL treatment. Similarly, TRAIL treatment of HeLa-CASP8 KO cells failed to activate apoptosis signaling, as assessed by PARP cleavage and flow cytometric analysis of annexin V/propidium iodide (PI) staining (Supplementary Fig. 1).

Initial experiments were conducted with a small subset of mutants (L105H, D303G, S386*, Q465*), then expanded to include all remaining mutants. The MT proteins were constructed as the proenzyme forms fused to a carboxyl-terminal FLAG epitope tag using a doxycycline (DOX)-inducible lentiviral vector (pLVX). FLAG-tagged WT caspase-8 proenzyme and FLAG-tagged LacZ were generated as positive and negative controls, respectively. The lentiviral constructs were used to infect HeLa-CASP8 KO cells and stably infected cells were selected in puromycin. Immunoblotting with anti-FLAG confirmed DOX-inducible expression of LacZ and the WT and MT caspase-8 proteins (Fig. 1B). As expected, truncation MT proteins (eg. S386*, S375*) exhibited reduced sizes.

**Fig. 2:**
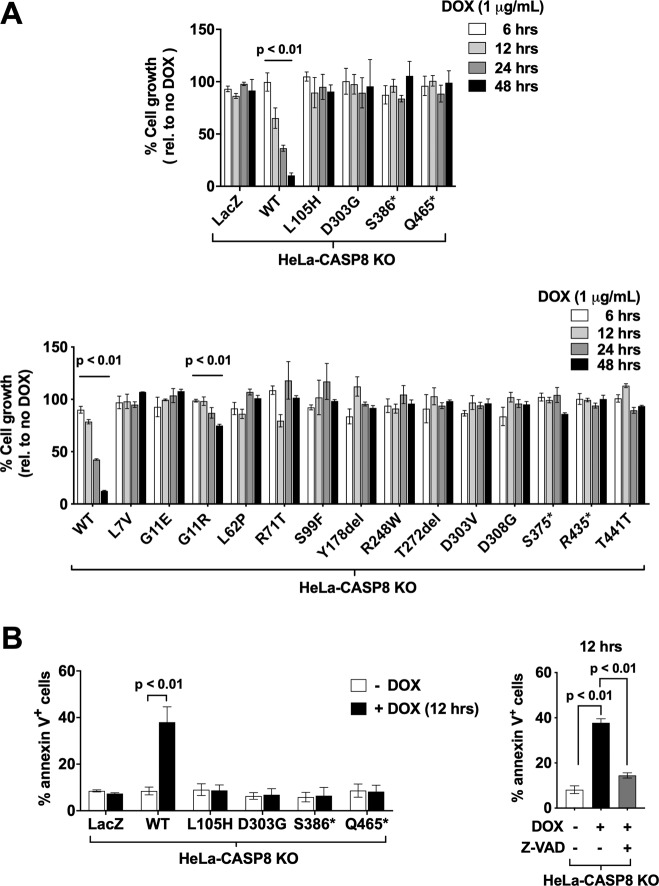
Exogenous expression of WT caspase-8, but not MT caspase-8 proteins, induces cell death. **A** HeLa-CASP8 KO cells engineered for DOX-inducible expression of WT caspase-8, MT caspase-8 proteins, or LacZ were treated in quadruplicate in the absence or presence of DOX (1 μg/mL) for varying lengths of time, followed by the performance of crystal violet (top panel) or MTT (bottom panel) assays. Data are expressed relative to no DOX treatment and one-way ANOVA was used to compare treatment groups. Columns represent the means from triplicate experiments; error bars represent the SEM. **B** In the left panel, cells were treated for 12 h in the absence or presence of DOX, followed by flow cytometric analysis of annexin V staining. Columns represent the average percentage of annexin V-positive cells from two independent experiments; error bars represent the SEM. Student’s *t* test was performed to compare groups with and without DOX induction. In the right panel, cells were treated in the absence or presence of DOX alone, or DOX plus z-VAD-FMK 30 μM) prior to analysis.

Prior studies have demonstrated that exogenous expression of WT caspase-8 zymogen induces cell death in the absence of death-ligand stimulation, presumably due to autoprocessing of the enzyme [25–27]. To determine whether the expression of the MT caspase-8 proteins would have the same effect, the isogenic cell lines were treated with DOX for varying lengths of time, followed by crystal violet or MTT assays to assess cell growth. Time-dependent inhibition of cell growth was detected in cells engineered for DOX-inducible expression of WT caspase-8, but not in cells engineered for the negative control, LacZ (Fig. 2A). No inhibition of cell growth was detected following DOX induction of the caspase-8 MT proteins, with the exception of cells expressing G11R, where a very modest, but statistically significant inhibition of cell growth was observed. Findings were confirmed by flow cytometric analysis of annexin V staining (Fig. 2B). DOX induction of WT caspase-8 led to a marked increase in annexin V staining, which was inhibited by the pan-caspase inhibitor z-VAD-FMK. In contrast, no increase in annexin V staining was seen following DOX induction of LacZ or four representative MT caspase-8 proteins. Taken together, these findings demonstrated that exogenous expression of HNSCC-associated caspase-8 MTs, unlike WT caspase-8, failed to induce cell death in the HeLa-CASP8 KO model.

### A subset of caspase-8 MTs retain the capacity to mediate TRAIL-induced cell death

**Fig. 3:**
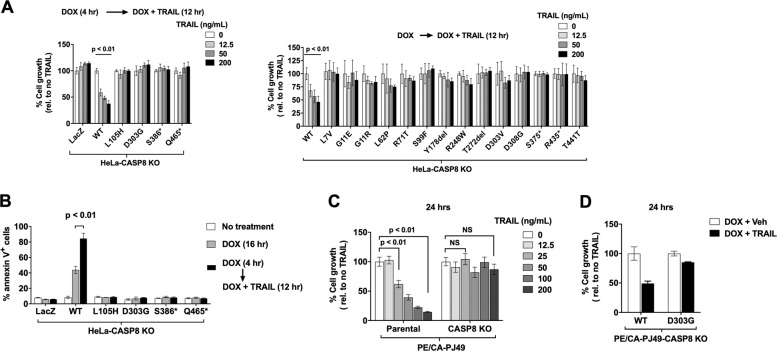
HNSCC-associated caspase-8 mutants fail to mediate TRAIL-induced apoptosis with similar potency to WT caspase-8. **A** HeLa-CASP8 KO cells engineered for DOX-inducible expression of WT caspase-8, MT caspase-8 proteins, or LacZ were pre-treated in quadruplicate with DOX (1 μg/mL), followed by treatment with DOX plus varying concentrations of TRAIL for an additional 12 h before the performance of crystal violet (upper panel) or MTT (lower panel) assays. Columns represent the means from quadruplicate wells; error bars represent the SEM. One-way ANOVA was used to compare multiple groups. Experiments were performed three times with similar results. **B** Cells were incubated for 16 h in the absence of treatment, for 16 h with DOX (1 μg/mL) alone, or for 4 h with DOX followed by 12 h with DOX plus TRAIL (200 ng/mL). Flow cytometry for annexin V staining was performed to evaluate the induction of apoptosis. Columns represent the average percentage of annexin V-positive cells from two independent experiments; error bars represent the SEM. Student’s *t* test was performed to compare differences between groups with and without TRAIL treatment. **C** Parental PE/CA-PJ49 cells or PE/CA-PJ49-CASP8 KO cells were treated for 24 h with the indicated concentrations of TRAIL, followed by the performance of MTT assays. Columns represent the means from eight wells; error bars represent SEM. Student’s *t* test was performed to compare groups without TRAIL treatment and with TRAIL at 25 ng/mL or 200 ng/mL. **D** PE/CA-PJ49-CASP8 KO cells engineered for DOX-inducible expression of WT caspase-8 or MT caspase-8 (D303G) were treated for 24 h with DOX plus vehicle (Veh) or DOX plus TRAIL (200 ng/mL), followed by the performance of MTT assays. Columns represent the means from quadruplicate wells; error bars represent the SEM.

We next examined the ability of individual MT caspase-8 proteins to mediate TRAIL induction of cell death. HeLa-CASP8 KO cells engineered for DOX-inducible expression of LacZ, WT caspase-8, or the MT caspase-8 proteins were pre-treated with DOX, followed by treatment with DOX plus varying concentrations of TRAIL for an additional 12 h (Fig. 3A). Cells expressing WT caspase-8 exhibited a dose-dependent inhibition of cell growth by TRAIL. By contrast, cells expressing LacZ or the MT caspase-8 proteins did not exhibit cell growth inhibition or apoptosis induction following 12-h TRAIL treatment (Fig. 3A, B).

To verify that our findings were not restricted to the HeLa-CASP8 KO model and could be validated in the context of a HNSCC model, we performed experiments using the TRAIL-sensitive HNSCC cell line PE/CA-PJ49 (Supplementary Fig. 2A). CRISPR was used to delete the expression of endogenous caspase-8 and the absence of WT caspase-8 expression in PE/CA-PJ49-CASP8 KO cells was confirmed by immunoblotting (Supplementary Fig. 2B). In contrast to the parental cell line, PE/CA-PJ49-CASP8 KO cells did not show growth inhibition following TRAIL treatment (Fig. 3C). PE/CA-PJ49-CASP8 KO cells engineered to express exogenous WT caspase-8, but not a representative MT caspase-8 (D303G; Supplementary Fig. 2C), demonstrated growth inhibition and apoptosis induction following TRAIL treatment (Fig. 3D and Supplementary Fig. 3), consistent with findings from the HeLa-CASP8 KO models.

**Fig. 4:**
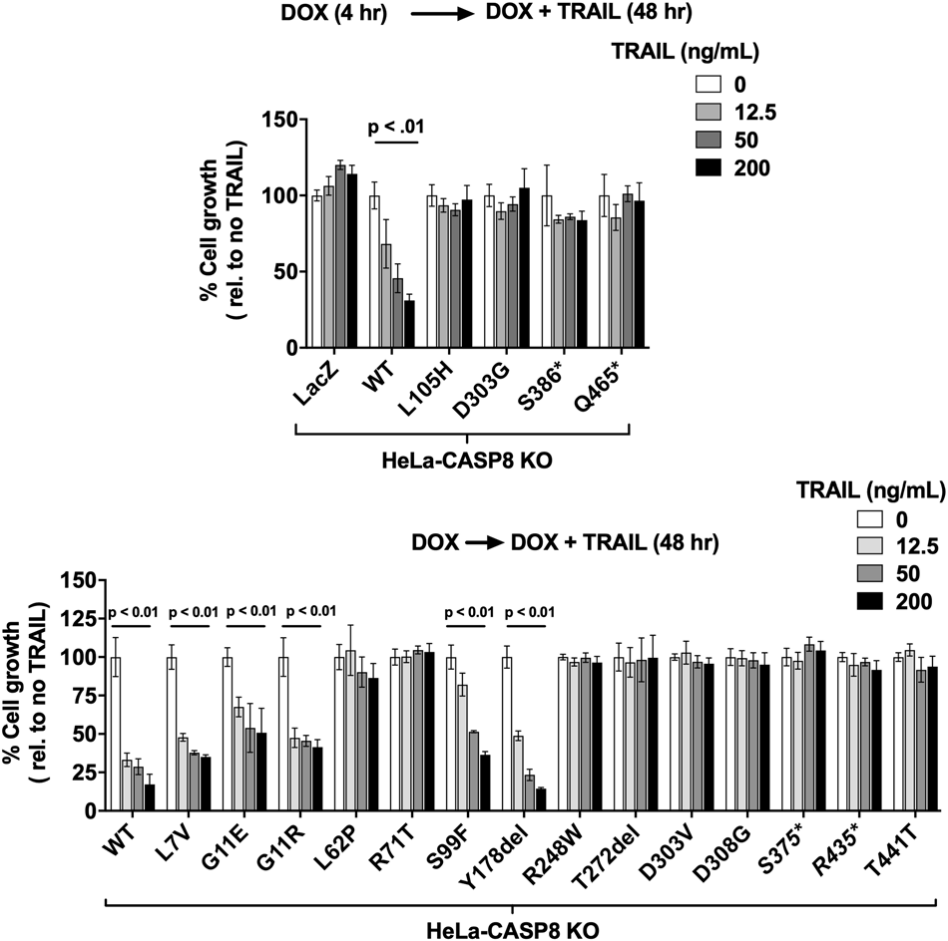
HNSCC-associated caspase-8 mutants display differential capacities to mediate TRAIL-induced cell death. HeLa-CASP8 KO cells engineered for DOX-inducible expression of WT caspase-8, MT caspase-8 proteins, or LacZ were pre-treated in quadruplicate with DOX (1 μg/mL), followed by treatment with DOX plus varying concentrations of TRAIL for an additional 48 h before the performance of crystal violet (upper panel) or MTT (lower panel) assays. Columns represent the means from quadruplicate wells; error bars represent the SEM. One-way ANOVA was used to compare multiple groups. Experiments were performed three times with similar results.

We next sought to determine whether the MT caspase-8 proteins might retain a weakened capacity to mediate TRAIL-induced cell death by lengthening the time of TRAIL treatment to 48 h. Notably, we observed that cells expressing five of the caspase-8 MT proteins (L7V, G11E, G11R, S99F, and Y178del) exhibited significant dose-dependent inhibition of cell growth after 48 h of treatment (Fig. 4). TRAIL induction of PARP cleavage was also detected in cells expressing the L7V, G11E, G11R, S99F, and Y178del mutants (albeit at reduced efficiency compared to cells expressing WT caspase-8), but not in cells expressing the other caspase-8 mutants (Supplementary Fig. 4A, B). Importantly, the five apoptosis-active mutants represent five of the eight mutations located in the amino-terminal prodomain of procaspase-8 (amino acids 1–181, numbering based on [28]), while none of the ten mutations in the carboxyl-terminal catalytic region (encoding the p18 and p10 subunits and the linker region between the subunits; amino acids 216–479 [29, 30]) were proficient at mediating apoptosis. For example, the carboxyl-terminal mutant D303G failed to mediate apoptosis after 48 h of TRAIL treatment in both the HeLa-CASP KO and PE/CA-PJ49-CASP8 KO models (Fig. 4 and Supplementary Fig. 3B).

### Carboxyl-terminal caspase-8 MTs retain the capacity to dimerize with WT caspase-8, while amino-terminal MTs are deficient

**Fig. 5:**
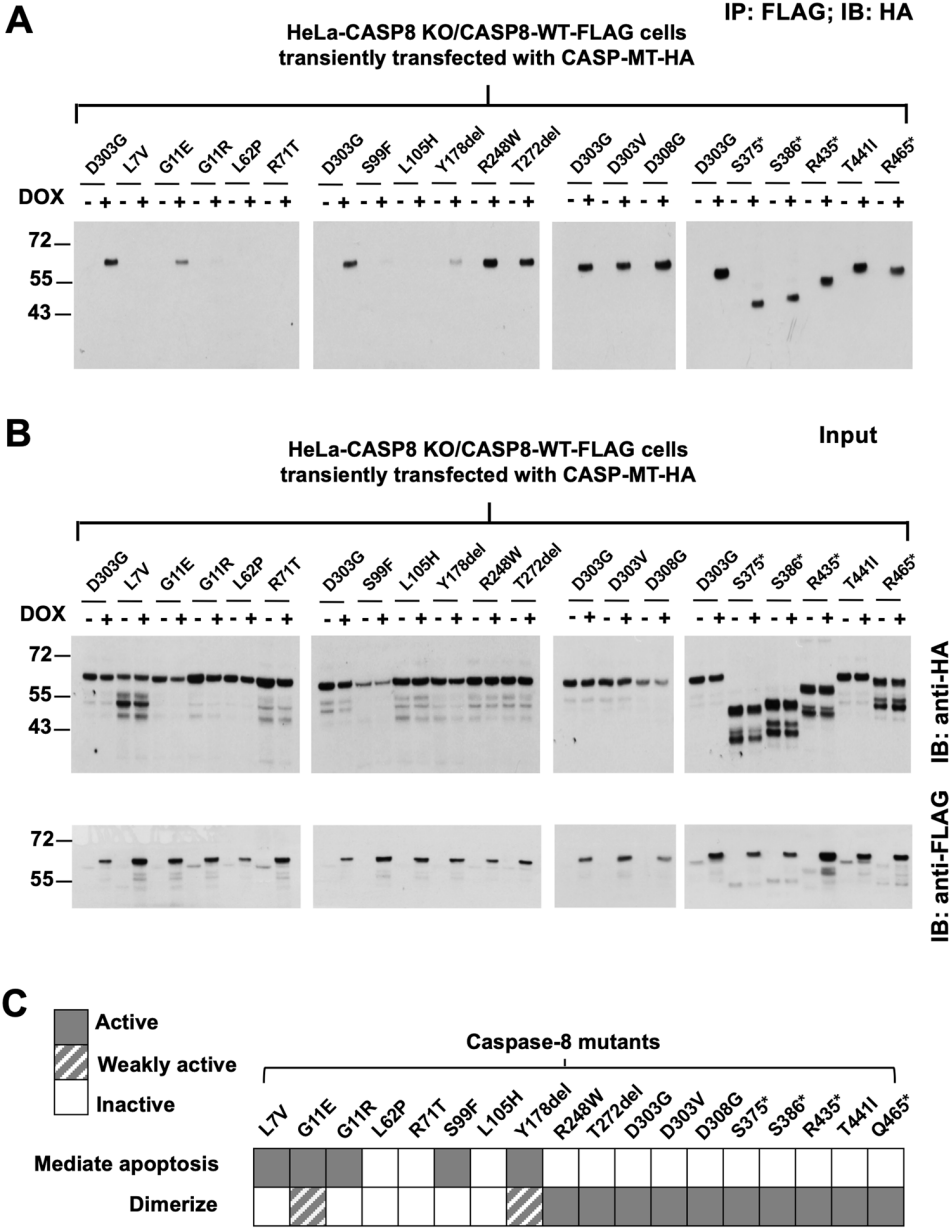
Carboxyl-terminal caspase-8 mutants efficiently dimerize with WT caspase-8 but are deficient in mediating TRAIL-induced apoptosis, while amino-terminal mutants fail to dimerize but are differentially effective at mediating apoptosis. Co-immunoprecipitations were performed to detect heterodimerization between MT caspase-8 proteins and WT caspase-8. **A** HeLa-CASP8 KO cells engineered for DOX-inducible expression of WT caspase-8-FLAG were treated in the absence or presence of DOX. Simultaneously, the cells were transiently transfected with constructs encoding the indicated HA-tagged caspase-8 mutants. Eight hours after transfection, cell lysates were prepared and subjected to immunoprecipitation with anti-FLAG, followed by immunoblotting with anti-HA to detect dimerization (for immunoprecipitations: 500 μg of input for D303G, L62P, R248W, T272del, D303V, T441I, R465*; 2000 μg of input for L7v, G11E, G11R, R71T, L105H, Y178del, D308G, R435*; 125 μg of input for S375* and S386*; 1750 μg of input for S99F). **B** Input lysates were analyzed by immunoblotting for expression of the MT caspase-8-HA and WT caspase-8-FLAG proteins. Due to varying levels of expression of the HA-tagged MT proteins, varying amounts of input lysate were loaded (25 μg for D303G, L62P, R248W, T272del, D303V, T441I, R465*; 100 μg for L7V, G11E, G11R, R71T, L105H, Y178del, D308G, R435*; 12.5 μg for S375* and S386*; 87.5 μg for S99F) Experiments in panels **A** and **B** were performed twice times with similar results. **C** The figure shows an inverse relationship for the caspase-8 mutants between the ability to mediate TRAIL-induced cell death and the capacity to heterodimerize with WT caspase-8.

TRAIL induction of apoptosis involves oligomerization-induced processing/activation of caspase-8. Caspase mutations that occur in HNSCC, and other cancers, typically occur in a heterozygous fashion. Thus, we performed co-immunoprecipitation (co-IP) assays to assess the ability of each caspase-8 MT to heterodimerize with WT caspase-8. HeLa-CASP8 KO cells engineered for DOX-inducible expression of WT caspase-8-FLAG were treated with DOX and simultaneously transfected with constructs encoding HA-tagged caspase-8 MT proteins (Fig. 5) or HA-caspase-8 WT protein (Supplementary Fig. 5). Immunoprecipitations were performed with anti-FLAG, followed by blotting with anti-HA (Fig. 5A). Immunoblotting of the input lysates was used to confirm the expression of the WT (anti-FLAG) and MT (anti-HA) caspase-8 proteins (Fig. 5B). In preliminary studies, we observed that the D303G MT was efficiently dimerized with WT caspase-8, so this positive control co-IP was loaded on every gel to permit comparisons between gels. As shown in Fig. 5A, proteins with mutations in the amino-terminal prodomain (L7V, G11E, G11R, L62P, R71T, S99F, L105H, Y178del) demonstrated little, if any, dimerization with WT caspase-8 (G11E and Y178del exhibited weak dimerization). Interestingly, this deficiency in dimerization was observed in all five of the amino-terminal caspase-8 MTs that retained a weak capacity to mediate TRAIL-induced cell death (Figs. 4 and 5A, C). By contrast, all ten proteins with mutations in the carboxyl-terminal catalytic region [R248W, T272-T273del (abbreviated as T272del), D303G, D303V, D308G, S375*, S386*, R435*, T441I, R465*] uniformly demonstrated efficient dimerization with WT caspase-8 (Fig. 5A). Of note, all of these dimerization-capable mutants lacked the ability to mediate TRAIL-induced cell death (Fig. 4). These findings demonstrated an inverse relationship between the ability of the HNSCC-associated caspase-8 mutants to dimerize with WT caspase-8 and their functional capacity to mediate TRAIL-induced cell death (Fig. 5C).

### HNSCC-associated caspase-8 mutants exhibit differential capacities to mediate TRAIL induction of immunosuppressive cytokines

**Fig. 6:**
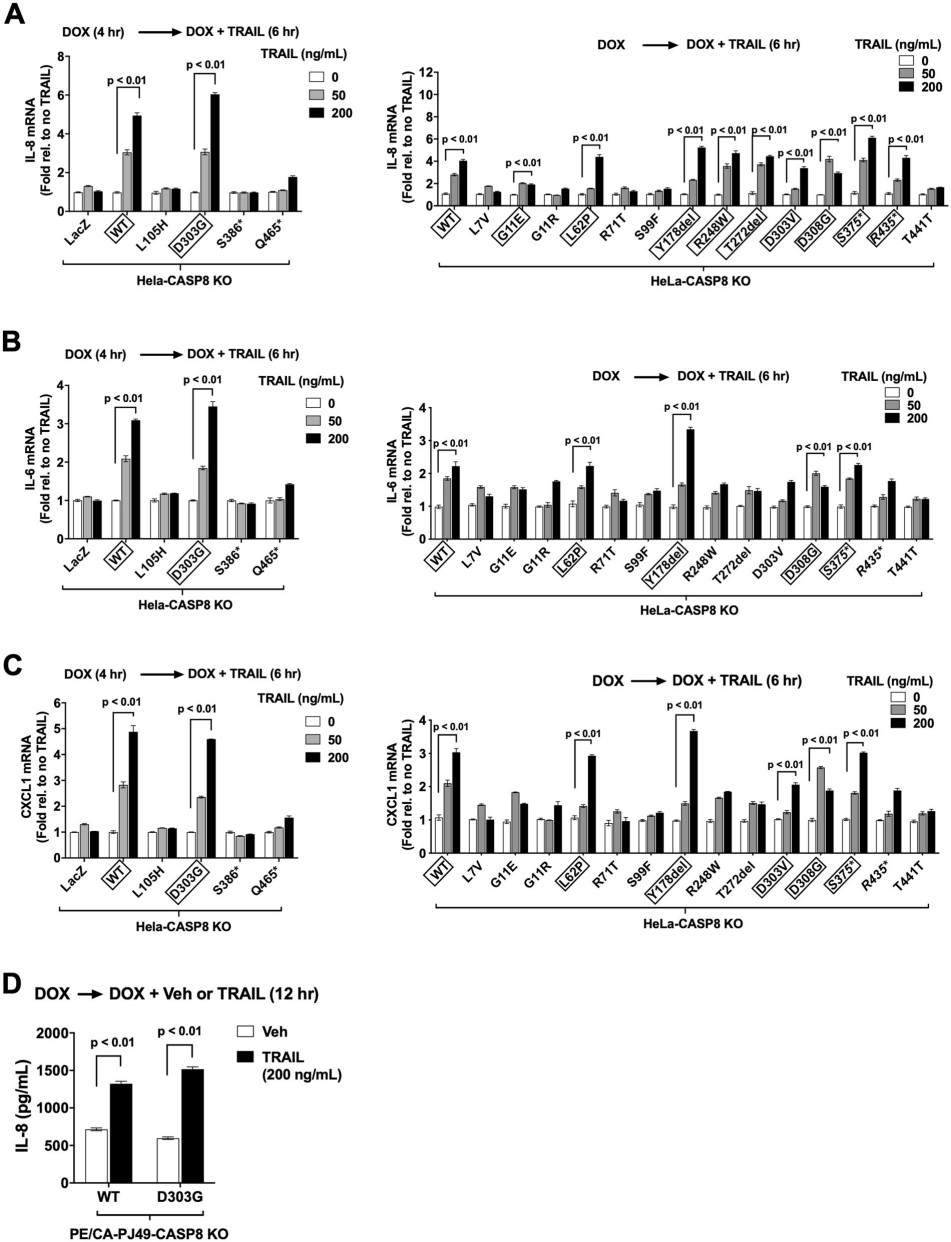
HNSCC-associated caspase-8 mutants exhibit differential capacities to mediate induction of IL-8, IL-6, and CXCL1. HeLa-CASP8 KO cells engineered for DOX-inducible expression of WT caspase-8, MT caspase-8 proteins, or LacZ, were pre-treated with DOX, followed by treatment for an additional 6 h with DOX plus varying concentrations of TRAIL. RNA was then harvested and qPCR was performed for IL-8 (**A**), IL-6 (**B**), and CXCL1 (**C**) mRNA levels. Data were plotted as the fold induction relative to no TRAIL. Columns represent the means from triplicate wells; error bars represent the SEM. Black rectangles outline WT or MT caspase-8 proteins that mediated twofold or greater induction. Student’s *t* test was performed on samples exhibiting twofold or greater induction. Experiments were performed three times with similar results. **D** PE/CA-PJ49-CASP8 KO cells engineered for DOX-inducible expression of WT caspase-8 or MT caspase-8 (D303G) were pre-treated with DOX, followed by treatment for 6 h with DOX plus vehicle or DOX plus TRAIL (200 ng/mL). Cell supernatants were harvested and ELISA assays were used to determine levels of secreted IL-8 protein. Columns represent the means from three independent experiments; error bars represent the SEM. Student’s *t* test was performed to compare groups treated with or without TRAIL.

Caspase-8 mediates death-ligand upregulation of specific cytokines/chemokines (e.g., IL-8, IL-6, CXCL1) via a mechanism that is independent of protease catalytic activity [4]. We confirmed that TRAIL treatment of parental HeLa cells upregulated mRNAs and protein for IL-8, IL-6, and CXCL1 in a dose-dependent fashion and that this effect was abrogated in HeLa-CASP8 KO cells (Supplementary Fig. 6A, B). We next tested whether the caspase-8 MT proteins might retain the ability to mediate TRAIL induction of cytokines. Cells were pre-treated with DOX, followed by treatment for an additional 6 h with DOX plus varying concentrations of TRAIL. As expected, cells exogenously expressing WT caspase-8 exhibited dose-dependent upregulation of IL-8, IL-6, and CXCL1 mRNAs in response to TRAIL treatment, while cells expressing LacZ did not (Fig. 6A–C). Ten of the caspase-8 mutants (shown in boxes) mediated a greater than twofold induction of IL-8 mRNA; 5 mutants (in boxes) mediated a greater than twofold induction of IL-6 mRNA; and six mutants (in boxes) mediated a greater than twofold induction of CXCL1 mRNA (Fig. 6A–C). Mutants that mediated greater than twofold induction of mRNAs for all three cytokines included L62P, Y178del, D303G, D308G, and S375*. In total, three of eight amino-terminal mutants and 9/10 carboxyl-terminal mutants mediated TRAIL induction of IL-8, IL-6, and/or CXCL1 mRNAs. ELISA-based measurement of secreted IL-8 confirmed TRAIL induction of cytokine protein in cells expressing a representative caspase-8 MT (D303G; Supplementary Fig. 7A, B). Taken together, our findings demonstrate that multiple HNSCC-associated caspase-8 mutants retain the ability to mediate TRAIL induction of immunosuppressive cytokines, and again make evident the differential functional properties of individual mutants.

Findings regarding cytokine upregulation were confirmed in the PE/CA-PJ49 HNSCC model. TRAIL treatment resulted in significant upregulation of IL-8 protein in parental PE/CA-PJ49 cells, an effect that was completely abrogated in PE/CA-PJ49-CASP8 KO cells (Supplementary Fig. 8). TRAIL induction of IL-8 protein was also observed in PE/CA-PJ49-CASP8 KO cells engineered for expression of either WT caspase-8 or the D303G MT caspase-8 (Fig. 6D), confirming the functional capacity of caspase-8 mutants to mediate cytokine induction by TRAIL.

### Inducible expression of caspase-8 impacts the tumor immune microenvironment in vivo

The impact of both WT and MT caspase-8 proteins on cytokine levels suggested that the expression of these proteins in tumor cells may alter the tumor microenvironment in vivo. Therefore, we assessed the effects of inducible expression of WT and MT caspase-8 on tumor immune profiles using an immunocompetent mouse model of HNSCC. For this purpose, we utilized MOC1 cells, which are derived from a C57BL/6 HNSCC tumor and form syngeneic tumors in wild-type C57BL/6 mice [31–33]. CRISPR was used to knock out endogenous *caspase-8* in MOC1 cells, generating MOC1-CASP8 KO cells. MOC1 cells were more refractory than HeLa or PE/CA-PJ49 to TRAIL, but were sensitive to the related death-ligand TNFα (Supplementary Fig. 9). Notably, TNFα induced cell death and IL-6 expression in parental MOC1 cells, but not in MOC1-CASP8 KO cells (Supplementary Fig. 9B–D). TNFα treatment also induced IL-6 in MOC1-CASP8 KO cells engineered for DOX-inducible expression of the D303G caspase-8 mutant, but not cells engineered for DOX-inducible LacZ (Supplementary Fig. 9E, F).

**Fig. 7:**
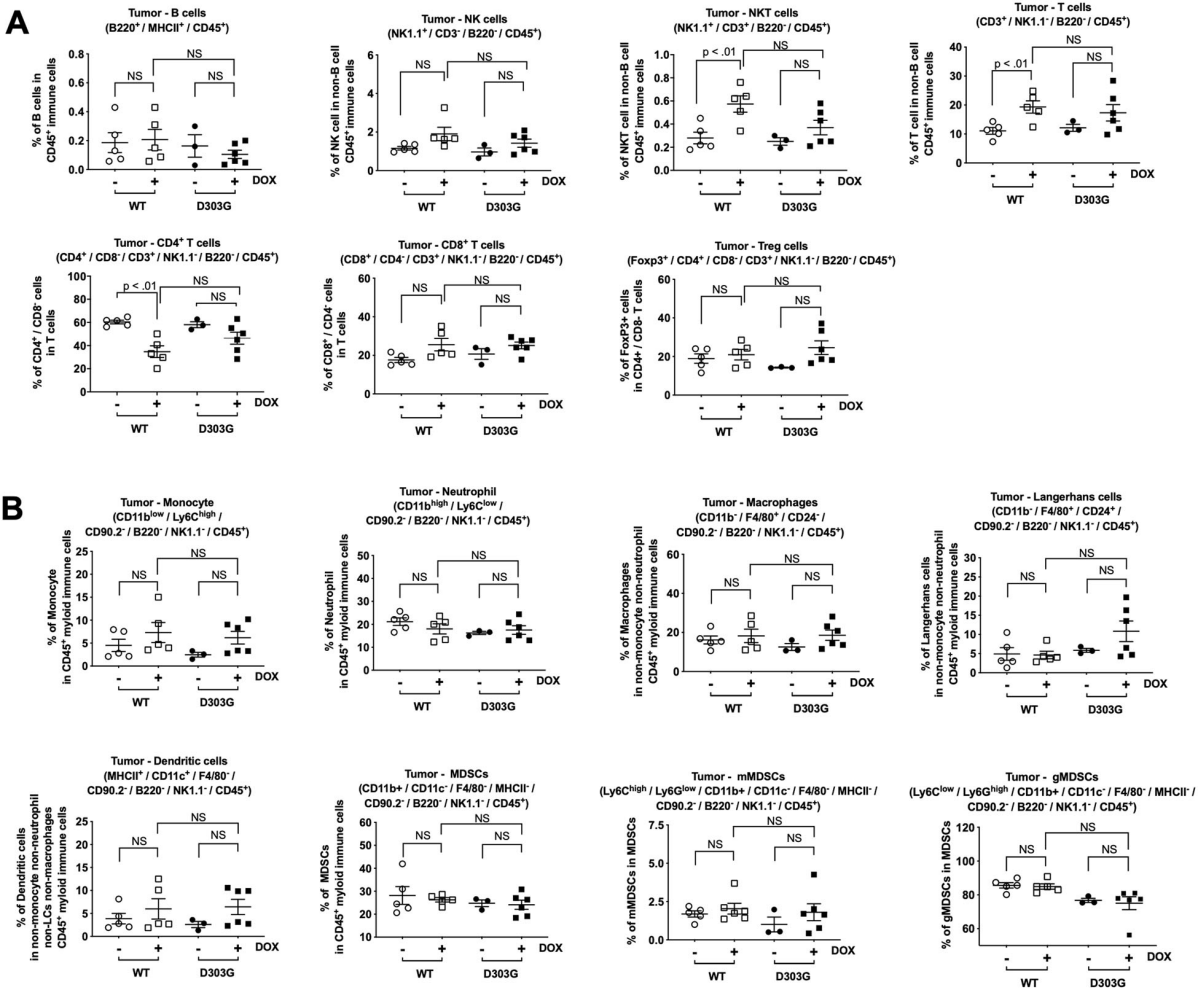
Impact of WT or MT caspase-8 expression on tumor immune microenvironment in vivo. MOC1-CASP8 KO cells engineered for DOX-inducible expression of WT caspase-8 or the D303G caspase-8 mutant were inoculated into the flanks of wild-type C57BL/6 mice. On day 28 (see Supplementary Fig. 8), mice were randomized to receive DOX (in drinking water) or no DOX. Tumors were harvested after 18 days of treatment and analyzed by flow cytometry for lymphoid (**A**) or myeloid (**B**) cell populations. In the DOX treatment groups, only tumors demonstrating DOX induction of the FLAG-tagged WT or MT caspase-8 (Supplementary Fig. 7) were analyzed. Error bars indicate SEM. Student’s *t* tests were performed to compare WT with DOX vs. WT without DOX; D303G with DOX vs. D303G without DOX, and WT with DOX vs. D303G with DOX.

MOC1-CASP8 KO cells engineered for DOX-inducible expression of murine versions of either WT caspase-8 or the D303G mutant were inoculated into the flanks of wild-type C57BL/6 mice and tumor-bearing mice were randomized to receive either DOX (in drinking water) or no DOX. Immunoblotting of tumor lysates revealed successful DOX induction of WT or MT caspase-8 tumors in most of the treated mice (Supplementary Fig. 10). Interestingly, DOX induction of WT or MT caspase-8 did not result in a statistically significant alteration of tumor growth (Supplementary Fig. 11). However, flow cytometric immunoprofiling of the tumors did reveal alterations in specific immune cell populations. Specifically, DOX induction of WT caspase-8 resulted in statistically significant increases in the percentages of T cells and NKT cells among the non-B cell immune cells (Fig. 7A). Among the T-cell population, the percentage of CD4+ was significantly reduced. Similar trends of increased percentages of T and NKT cells, and reduced CD4+ cells in the T-cell population, were detected in tumors following DOX induction of the D303G caspase-8 mutant, although these trends did not achieve statistical significance. No statistically significant alterations in myeloid cell populations were detected following induction of either WT or MT caspase-8 (Fig. 7B). Taken together, these findings indicate that expression of WT or MT caspase-8 by HNSCC tumor cells alters the tumor immune microenvironment.

## Discussion

Cancer-associated mutations in procaspase-8 are widely distributed throughout the protein-coding region and are commonly assumed to represent the loss of functional proteins. Approximately 10% of human HNSCC tumors exhibit mutations in procaspase-8 [19]. In this study, we tested the hypothesis that HNSCC-associated procaspase-8 mutants are loss of function proteins, by investigating whether the mutant proteins retain any of the functional and/or phenotypic properties associated with WT procaspase-8. Our studies determined that mutants in the carboxyl-terminal catalytic region retained the ability to dimerize with WT caspase-8, while mutants in the amino-terminal prodomain region were largely deficient in dimerization. On the other hand, carboxyl-terminal mutants lacked the ability to mediate TRAIL-induced apoptosis, while over half (5/8) of the amino-terminal mutants retained the ability to mediate apoptosis, albeit with reduced efficiency relative to WT. Several mutants in either the amino-terminal or carboxyl-terminal regions mediated TRAIL induction of IL-8, IL-6, and/or CXCL1, in a fashion similar to WT caspase-8. Thus, our findings demonstrate that HNSCC-associated caspase-8 mutants are not simple loss of function proteins, but differentially retain distinct functional properties found in WT caspase-8. Our in vivo findings with a representative chemokine/cytokine-inducing mutant (D303G) suggest that some caspase-8 mutants, like WT caspase-8, may impact the immune composition of the HNSCC tumor microenvironment, presumably via modulation of the chemokine/cytokine milieu.

A few limitations of our study should be noted. Although studies were performed in three different models (HeLa, PE/CA-PJ49, MOC1), most were done in HeLa, an imperfect model, as it is an HPV-positive cervical cancer cell line. MOC1 cells harbor a caspase-9 mutation; thus, type II apoptosis may be blocked in this model. There is also concern with the use of knockout models that the genetics of the knockout line may differ markedly from that of the parental cells. Lastly, our immune studies are preliminary in nature and only allow us to conclude that the tumor microenvironment differs with the expression of WT or MT caspase-8. Future studies will be needed to more precisely assess the impact on specific immune cell populations.

While caspase-8 mutations occur most commonly in HNSCC (10%), they have also been reported in multiple other malignancies, including uterine (9.8%), stomach (4.8%), cervical (4.4%), colorectal (3.5%), melanoma (3.4%), and bladder (3.2%) cancers (TCGA, PanCancer Atlas [18]). Although strong hotspot mutations are not evident, mutations at several sites are reported in tumors from more than one HNSCC patient, as well as more than one cancer type. For example, mutations at R68 have been reported in tumors from 13 different patients, including patients with breast, lung, uterine, and HNSCC cancers; mutations at D303 have been detected in lung cancer and HNSCC. The recurrence of caspase-8 mutations at specific sites in multiple cancers, argues that these mutations contribute to malignant progression.

In an effort to selectively focus on the properties of mutant caspase-8 proteins without the confounding presence of WT caspase-8, we engineered DOX-inducible expression of the mutants in cell lines wherein the WT *caspase-8* gene had been deleted. However, it should be noted that HNSCC-associated caspase-8 mutations are heterozygous [19], and the presence of the WT protein may bring to light additional properties of the mutants. Particularly likely is the possibility that the mutant proteins act in a dominant-negative fashion. Indeed, a prior study found that L105H, S375*, and S386*, three of the mutants investigated in the current study, inhibit death-ligand-induced apoptosis when co-expressed with WT caspase-8 [27]. As our studies show that 10/10 carboxyl-terminal mutants retain strong dimerization with WT protein, yet are entirely deficient in mediating apoptosis, the mechanistic underpinnings suggest a dominant-negative impact of these mutants on the WT caspase-8 protein.

Human HNSCC cell lines with endogenous mutations in caspase-8 are more tumorigenic, relative to HNSCC cell lines with WT caspase-8, when grown as orthotopic tumors in athymic nude mice [22]. We investigated the impact of both WT caspase-8 and a representative mutant (D303G) on the growth of HNSCC tumors in an immunocompetent environment using the MOC1 HNSCC model [31–33]. Notably, tumor growth in this model was not impacted following induction of either the WT or MT protein. These discordant findings could be due to complex genetic differences in the human cell lines that were previously tested [22], differences between immunodeficient and immunocompetent mouse models, or the use of orthotopic versus subcutaneous flank tumors.

Lastly, future efforts to develop therapies that potently eliminate caspase-8 mutant tumors should focus on identifying the specific vulnerabilities of these tumor cells. Synthetic lethal screens may be particularly helpful. It is noteworthy that the loss of caspase-8 activity can serve to activate necroptosis [34, 35]. Recent studies have shown that knockdown of caspase-8 enhances the sensitivity of HNSCC tumors to Birinapant, a second mitochondria-derived activator of caspase (SMAC) mimetic and necroptosis sensitizer [24]. Future studies are needed to determine whether HNSCC tumors harboring caspase-8 mutations are similarly sensitized to Birinapant or other necroptosis inducers.

## Materials and methods

### Cell lines and reagents

HeLa cells were obtained from American Type Culture Collection (ATCC; Rockville, MD, USA) and PE/CA-PJ49 cells from Sigma-Aldrich. Both cell lines were cultured in Dulbecco’s Modified Eagle’s Medium (DMEM; Mediates Inc.) with 10% fetal bovine serum (FBS; Gemini Bioproducts) and 1% penicillin and streptomycin (Life Technologies). MOC1 cells [31–33] were the kind gift of Ravi Uppaluri (Harvard), and was cultured in IMDM (Gibco)/HAM’s F-12 (HyClone) at a 2:1 ratio and supplemented with 5% FBS, 1% penicillin/streptomycin, EGF (5 ng/ml), hydrocortisone (400 ng/ml), and insulin (5 mg/ml). All cell lines were authenticated via short tandem repeat (STR) testing (Genetica DNA Laboratories) and tested for mycoplasma using the PlasmoTest Kit (InvivoGen).

### Generation of caspase-8 knockout cells

The endogenous *caspase-8* gene in HeLa and PE/CA-PJ49 cells was knocked out using CRISPR Cas9 technology. pSpCas9(BB)-2A-GFP (pX458) was a gift from Feng Zhang (Addgene plasmid # 48138). Guide RNA oligos (forward: 5′-CACCGACAACTGGACAGTGAAGATC-3′; reverse: 3′-AAACGATCTTCACTGTCCAGTTGTC-5′) were inserted into the pX458 vector and constructs transfected into cells using Lipofectamine 2000 (Invitrogen). Limiting dilution was used to obtain single-cell CASP8 KO clones.

### Generation of stable cells with inducible expression of WT or MT caspase-8

WT and mutated caspase-8 cDNA sequences were cloned into the DOX-inducible lentiviral vector pLVX-TetOne-CminDEST (pLVX), a kind gift from Nevan Krogan (UCSF). Caspase-8 cDNAs (WT and MT) were first cloned into the entry vector pDONR221 (ThermoFisher #12536017) using BP clonase (Invitrogen). LR clonase (Invitrogen) was used to move the insert into pLVX. Lentiviral particles were produced by co-transfecting the constructs with helper plasmids (Envelope proteins VSV-G and Packaging proteins Gag-Pol-Tat-Rev) into HEK293T cells using FuGENE HD (Promega). Target cells were transduced by virus particles in the presence of polybrene (Santa Cruz Biotechnology) followed by selection in puromycin (2 μg/mL).

### Viability assays and annexin V staining

Cells were seeded in 96-well plates at 5000 cells/well in 100 μL of complete medium. The following day, cells were treated with DOX or TRAIL (AdipoGen, AG-40T-0002). Cell survival was examined by MTT (3-(4, 5-dimethylthiazol-2-yl)-2, 5-diphenyltetrazolium bromide) or crystal violet assays. For Annexin V staining, treated cells (5 × 10^5^) were harvested, resuspended in Annexin V Binding Buffer (BD, 556454), and Annexin V—AF488 antibody (2 μL, BD, 51-65874x) was added for 15 min at room temperature in the dark. Cells were then analyzed using a BD FACS Calibur DXP8 flow cytometer.

### Immunoblotting

Primary antibodies used: anti-caspase-8 (#4790S), anti-PARP (#9542S) and anti-GAPDH (#5174S) from Cell Signaling Technology, anti-HA (Novus, NB600-363), and anti-FLAG M2 (#F3165) from Sigma-Aldrich. Immunoblotting was performed as previously described [36].

### Transient transfections and co-immunoprecipitations

For co-IPs, expression constructs were generated encoding MT caspase-8 proteins tagged at the carboxyl terminus with HA epitope. Constructs (10 μg) were transiently transfected into HeLa-CASP8 KO cells stably expressing DOX-inducible WT caspase-8-FLAG for 8 h using lipofectamine 2000 (ThermoFisher Scientific, 11668019). Simultaneously, DOX (1 μg/mL) was added to induce WT caspase-FLAG expression. After 8 h, cells were lysed in IP lysis buffer (50 mM Tris-HCL pH 7.5; 150 mM NaCl; 1% Nonidet P-40) and aliquots of the input material were analyzed by immunoblotting with anti-FLAG or anti-HA. For immunoprecipitation, lysates were mixed with 30 μL anti-FLAG M2 beads slurry (Sigma, M8823) and incubated at 4 °C overnight. The beads were then washed three times in IP washing buffer (50 mM Tris-HCL pH 7.5; 150 mM NaCl; 0.05% Nonidet P-40), resuspended in sample buffer, and processed for immunoblotting.

### ELISA

Cells in six-well plates were treated with DOX for 4 h, followed by DOX in the absence or presence of TRAIL. Cell medium was collected and centrifuged to remove cellular debris. Human IL-8 (DY208, DuoSet, R&D Systems), IL-6 (DY206, DuoSet, R&D Systems), and CXCL1 (DY275, DuoSet, R&D Systems) ELISA assays were performed according to the manufacturer’s instructions, normalizing readings to the manufacturer’s standard.

### Real-time quantitative PCR

RNeasy kit (Qiagen) was used for total RNA isolation. Total RNA (2 μg) was used to make cDNA using the iScript Reverse Transcription Supermix Kit (Bio-Rad Laboratories). RT-qPCR was conducted using the CFX96 Real-Time System C1000 Touch Thermal Cycler (Bio-Rad Laboratories). PCR reactions were performed using SYBR Green Master Mix (Applied Biosystems #4367659). 2^-ΔΔCt^ values were compared between target genes and GAPDH to normalize values.

### Tumor growth and flow cytometric analyses

Female wild-type C57BL/6 mice (Jackson Labs) were inoculated in the right flank with 3 × 10^6^ MOC1-CASP8 KO cells engineered for DOX-inducible expression of murine versions of WT caspase-8 or the D303G MT caspase-8. When tumors reached 200 mm^3^, mice were randomized to receive drinking water containing DOX (0.5 mg/mL) plus sucrose (5 mg/mL) or drinking water with sucrose alone. Tumor volumes were determined three times per week.

After 18 days of DOX treatment, all mice were sacrificed, and tumors were harvested for flow cytometric analysis. Tumor cells were isolated by collagenase IV (4 mg/mL) and DNase I (0.4 mg/mL) treatment and filtered through a 70-μm strainer. Single cells (1.5 × 10^6^) were stained with lymphoid and myeloid cocktail antibodies. Zombie NIR (Biolegend, 423106) was used for the delineation of viable cells. Cell surface marker antibodies were all from Biolegend. CD45 (103151) antibody was used for the detection of immune cells. A cocktail of antibodies for Ly6G (127625), CD11b (101254), CD24 (101822), CD103 (121414), MHCII (107620), F4/80 (123133), Ly6C (128037), CD90.2 (105331), NK1.1 (108749), and B220 (103246) was used for identification of myeloid cells. A cocktail of antibodies for B220 (103236), CD8 (100758), CD4 (100422), CD3 (100236), MHCII (107620), and NK1.1 (108749) was used to identify lymphoid cells. FoxP3 (126406) antibody staining used the Foxp3/Transcription Factor Staining Buffer Set (eBioscience). Stained cells were detected using a 4-laser, 14-color Attune NxT flow cytometer (Thermo Scientific), and immune profiles were analyzed using the FlowJo V10 software (FlowJo LLC).

## Supplementary information

Supplementary Figure Legends

Supplementary Figure 1A and 1B

Supplementary Figure 2A, 2B, and 2C

Supplementary Figure 3A and 3B

Supplementary Figure 4A and 4B

Supplementary Figure 5A and 5B

Supplementary Figure 6A and 6B

Supplementary Figure 7A and 7B

Supplementary Figure 8

Supplementary Figure 9A, 9B, 9C, 9D, 9E, and 9F

Supplementary Figure 10

Supplementary Figure 11
