# Supplementary material for: Caspase-8 mutations associated with head and neck cancer differentially retain functional properties related to TRAIL-induced apoptosis and cytokine induction
Source: Cell Death Dis. 2021 Aug 6;12(8):775. doi: 10.1038/s41419-021-04066-z (PMC8346537; doi:10.1038/s41419-021-04066-z)
Supplement: Supplementary file 1 — Supplementary Figure Legends [file 41419_2021_4066_MOESM1_ESM.pdf]

Supplementary Figure Legends

Supplementary Fig. 1 Genetic deletion of *caspase-8* abrogates TRAIL induction of HeLa cell death. **A** Parental HeLa and HeLa-CASP8 KO cells were treated for 24 hours in the absence or presence of TRAIL (200 ng/mL), followed by immunoblotting for PARP or GAPDH. Similar results were seen in three independent experiments. **B** Parental HeLa and HeLa-CASP8 KO cells were treated for 24 hours in the absence or presence of varying concentrations of TRAIL, followed by flow cytometric analysis of annexin V or propidium iodide (PI) staining.

Supplementary Fig. 2 Generation of models in the HNSCC cell line PE/CA-PJ49. **A** PE/CA-PJ49, a HNSCC cell line, was treated for 24 or 48 hours with the indicated concentrations of TRAIL, followed by performance of MTT assays. Data were plotted as the percent of cell growth relative to no TRAIL treatment. Columns represent the means from quadruplicate wells; error bars represent the SEM. One-way ANOVA was performed for comparison of differences between multiple groups. **B** Parental PE/CA-PJ49 cells and PE/CA-PJ49-CASP8 KO cells were subjected to immunoblotting for caspase-8 or GAPDH. **C** PE/CA-PJ49-CASP8 KO cells engineered for DOX-inducible expression of FLAG-tagged, WT caspase-8 or MT caspase-8 (D303G), were treated for 24 hours in the absence or presence of DOX (1 μg/mL), followed by immunoblotting with anti-FLAG. Immunoblotting for GAPDH was used as a loading control.

Supplementary Fig. 3 HNSCC-associated caspase-8 mutant fails to mediate TRAIL-induced apoptosis with similar potency to WT caspase-8 in PE/CA-PJ49 HNSCC cells. A PE/CA-PJ49-CASP8 KO cells engineered for DOX-inducible expression of WT caspase-8 or MT caspase-8 (D303G) were treated for 24 hours with no DOX, DOX alone, or DOX plus TRAIL, followed by flow cytometric analysis of annexin V and PI staining. **B** Cells were treated for 48 hours with DOX or DOX plus TRAIL before performance of MTT assays.

Supplementary Fig. 4 HNSCC-associated caspase-8 mutants display differential capacities to mediate TRAIL induced PARP cleavage. A HeLa-CASP8 KO cells engineered for DOX inducible expression of WT caspase-8 or the indicated caspase-8 mutants were pre-treated for 2 hours with DOX, followed by treatment with DOX plus vehicle (V) or DOX plus TRAIL (T; 200 ng/mL) for an additional 6 or 24 hours. Immunoblotting was performed to detect total and cleaved PARP. Blotting for GAPDH was used as a loading control. To allow comparison of gels, an equivalent amount of lysate from cells expressing exogenous WT caspase-8 was loaded in the left lanes of each gel. **B** Densitometry was performed on the immunoblotting data in panel A to allow determination of the cleaved PARP/GAPDH ratio for each sample. Columns represent the means of triplicate densitometric values calculated by ImageJ and error bars represent SEM. The experiments were performed 3 times with similar results.

Supplementary Fig. 5. Homodimerization of WT CASP8-FLAG/WT CASP8-HA compared to heterodimerization of WT CASP8-FLAG/MT(D303G) CASP8-HA. Co-

immunoprecipitations were performed to detect homodimerization of WT caspase-8 and heterodimerization between MT caspase-8 proteins and WT caspase-8. **A** HeLa-CASP8 KO cells engineered for DOX-inducible expression of WT caspase-8-FLAG were treated in the absence or presence of DOX. Simultaneously, the cells were transiently transfected with constructs encoding the indicated WT caspase-8-HA or MT caspase-8-HA. Experiments were performed in the presence of 30 μM z-VAD-FMK to minimize loss of caspase-8 proteins due to processing. Eight hours after transfection, cell lysates were prepared and subjected to immunoprecipitation with anti-FLAG, followed by immunoblotting with anti-HA to detect dimerization (for immunoprecipitations: 500 μg of input for WT; 375 μg for D303G). **B** Input lysates were analyzed by immunoblotting for expression of the MT caspase-8-HA and WT caspase-8-FLAG proteins. Due to varying levels of expression of the HA-tagged MT proteins, varying amounts of input lysate were loaded (50 μg for WT; 37.5 μg for D303G) Experiments in panels A and B were performed three times with similar results.

Supplementary Fig. 6 Genetic deletion of caspase-8 abrogates TRAIL induction of cytokine mRNAs and proteins in HeLa cells. A Parental HeLa cells were treated for 6, 12, or 24 hours with the indicated concentrations of TRAIL. Following treatment, RNA was harvested and subjected to qPCR for IL-8, IL-6, and CXCL1 mRNAs. Data were plotted as the fold induction relative to that observed in cells that received no TRAIL. Columns represent the means from triplicate wells; error bars represent the SEM. The experiment was performed 3 times with similar results. **B** Parental HeLa cells and HeLa-CASP8 KO cells were treated for 24 hours with vehicle or TRAIL (200

ng/mL). Cell supernatants were harvested and ELISA assays were performed to assess IL-8, IL-6, and CXCL1 protein levels in TRAIL treated cells relative to vehicle treated cells. Data were plotted as the fold induction relative to no TRAIL. Columns represent the means from triplicate wells; error bars represent the SEM. Similar results obtained in two independent experiments.

Supplementary Fig. 7 The D303G caspase-8 MT mediates TRAIL induction of IL-8 protein. A HeLa-CASP8 KO cells engineered to express DOX inducible LacZ or WT caspase-8 were pre-treated with DOX for 4 hours, followed by treatment for an additional 12 hours with DOX plus vehicle or DOX plus TRAIL (200 ng/mL). Cell supernatants were harvested and ELISA assays were used to determine levels of secreted IL-8 protein. Columns represent the means from triplicate wells; error bars represent the SEM. Student's *t* test was performed to compare groups treated with or without TRAIL. Similar results were obtained in 3 independent experiments. **B** HeLa-CASP8 KO cells engineered to express DOX inducible D303G caspase-8 were pre-treated with DOX, followed by treatement for 6 or 12 hours with DOX plus vehicle or DOX plus TRAIL in medium lacking serum. IL-8 levels in supernatants were measured by ELISA. Columns represent the means from 3 independent experiments; error bars represent the SEM. Student's *t* test was performed to compare groups treated with or without TRAIL.

Supplementary Fig. 8 Caspase-8 is required for TRAIL induction of IL-8 protein in PE/CA-PJ49 cells. Cells were treated for 24 hours with vehicle or TRAIL (200 ng/mL)

and supernatants were collected and analyzed by ELISA for secreted IL-8 protein. Columns represent the means from triplicate wells; error bars represent the SEM. Student's *t* test was performed to compare groups treated with vehicle or TRAIL. Similar results obtained in 3 independent experiments.

Supplementary Fig. 9. **Caspase-8 mediates death ligand induction of cell death and cytokine expression in MOC1 cells.** **A** Parental MOC1 cells and MOC1-CASP KO cells were subjected to immunoblotting with anti-caspase-8. Immunoblotting with anti-GAPDH was used as loading control. **B** MOC1 cells were treated for 48 hours in the absence or presence of TRAIL (200 ng/mL), followed by performance of MTT assays. **C** Parental MOC1 cells and MOC1-CASP8 KO cells were treated in the absence or presence of TNFα (200 ng/mL), followed by MTT assays. **D** Cells were treated in the absence or presence of TNFα (200 ng/mL), followed by RT-qPCR for IL-6 mRNA. **E** MOC1-CASP8 KO cells engineered for DOX-inducible expression of FLAG-tagged LacZ, WT caspase-8, or the D303G caspase-8 MT, were treated in the absence or presence of DOX, followed by immunoblotting with anti-FLAG or anti-GAPDH. **F** MOC1-CASP8 KO cells engineered for DOX-inducible expression of LacZ or the D303G caspase-8 MT were treated for 16 with DOX, followed by 12 hours of treatment with DOX plus TNFα and RT-qPCR for IL-6 mRNA.

Supplementary Fig. 10 DOX inducible expression of WT or MT caspase-8 in MOC1 tumors in immunocompetent mice. MOC1 tumors engineered for DOX inducible expression of WT caspase-8 or the D303G caspase-8 mutant were grown in

wild-type C57BL/6 mice and treated with either DOX or no DOX. Tumors harvested at the end of treatment were subjected to immunoblotting with anti-FLAG to detect expression of the induced proteins. The tumor samples which demonstrated DOX-induced expression of WT (mouse #1, 4, 6, 7, 8) or MT caspase-8 (#9, 10, 11, 13, 14, 16) were used for plotting of growth curves (Supplementary Fig. 8) or immune profiling (Fig. 7A and 8B).

Supplementary Fig. 11 Growth curves for MOC1 tumors engineered for DOX inducible expression of WT caspase-8 (open symbols) or the D303G caspase-8 mutant (closed symbols). Treatment of tumor-bearing mice with DOX (squares), or no DOX (circles) was initiated at the indicated time point. Tumor volume was determined thrice weekly. In the DOX treatment groups, only tumors demonstrating DOX induction of the FLAG-tagged WT or MT caspase-8 (Supplementary Fig. 7) were plotted.
