# Supplementary figures and images for: Caspase-8 mutations associated with head and neck cancer differentially retain functional properties related to TRAIL-induced apoptosis and cytokine induction
Source: Cell Death Dis. 2021 Aug 6;12(8):775. doi: 10.1038/s41419-021-04066-z (PMC8346537; doi:10.1038/s41419-021-04066-z)

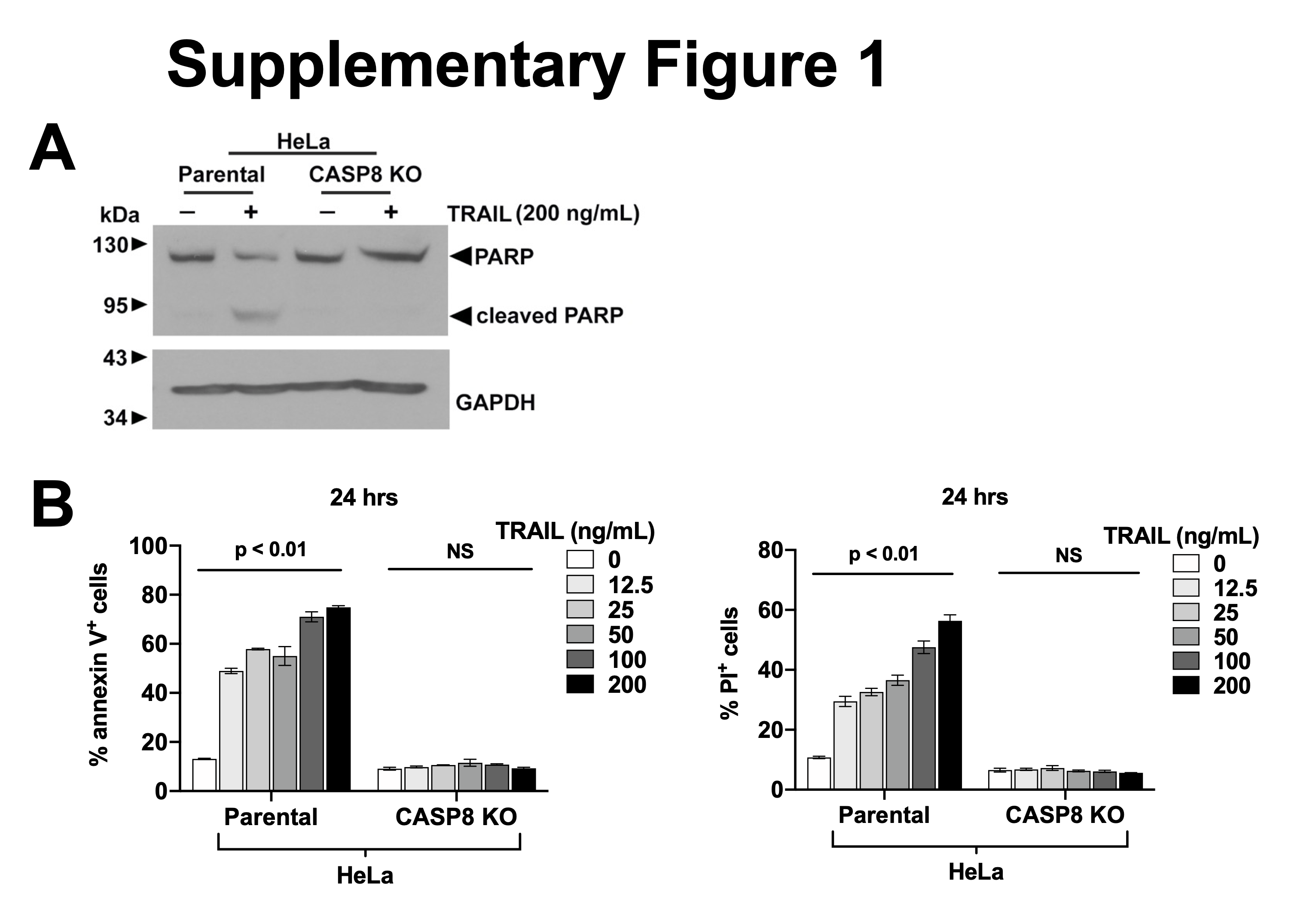

Supplement: Supplementary file 2 — Supplementary Figure 1A and 1B [file 41419_2021_4066_MOESM2_ESM.tif]

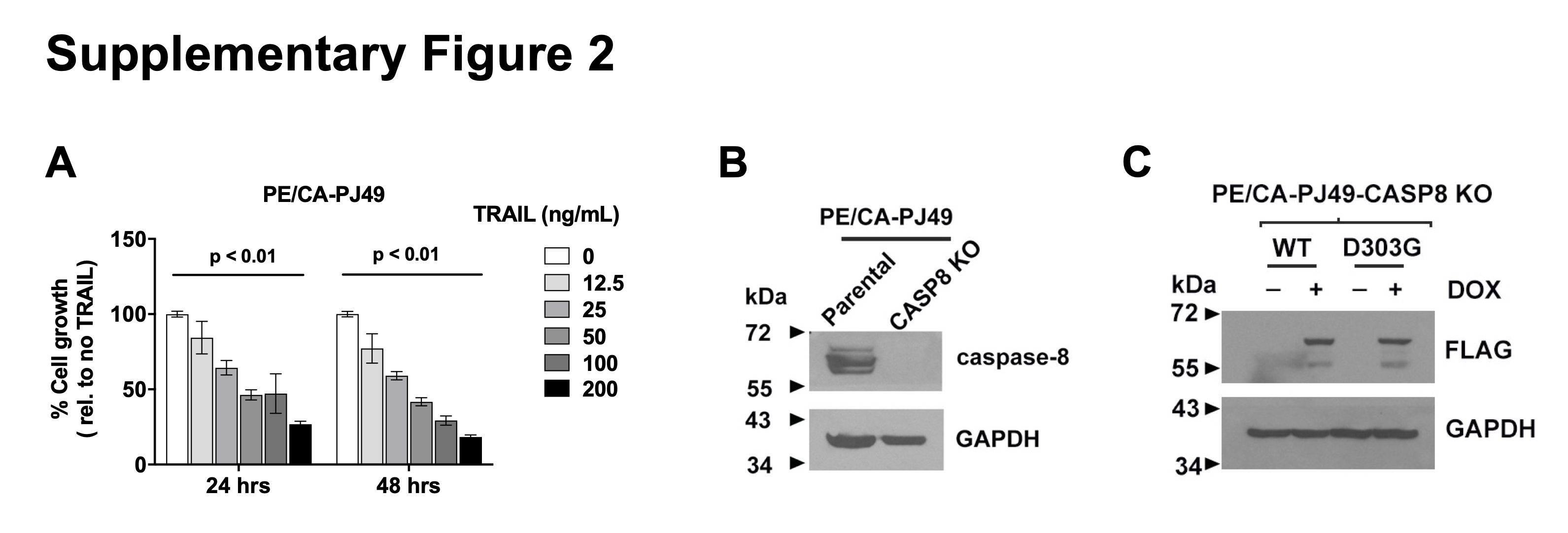

Supplement: Supplementary file 3 — Supplementary Figure 2A, 2B, and 2C [file 41419_2021_4066_MOESM3_ESM.tif]

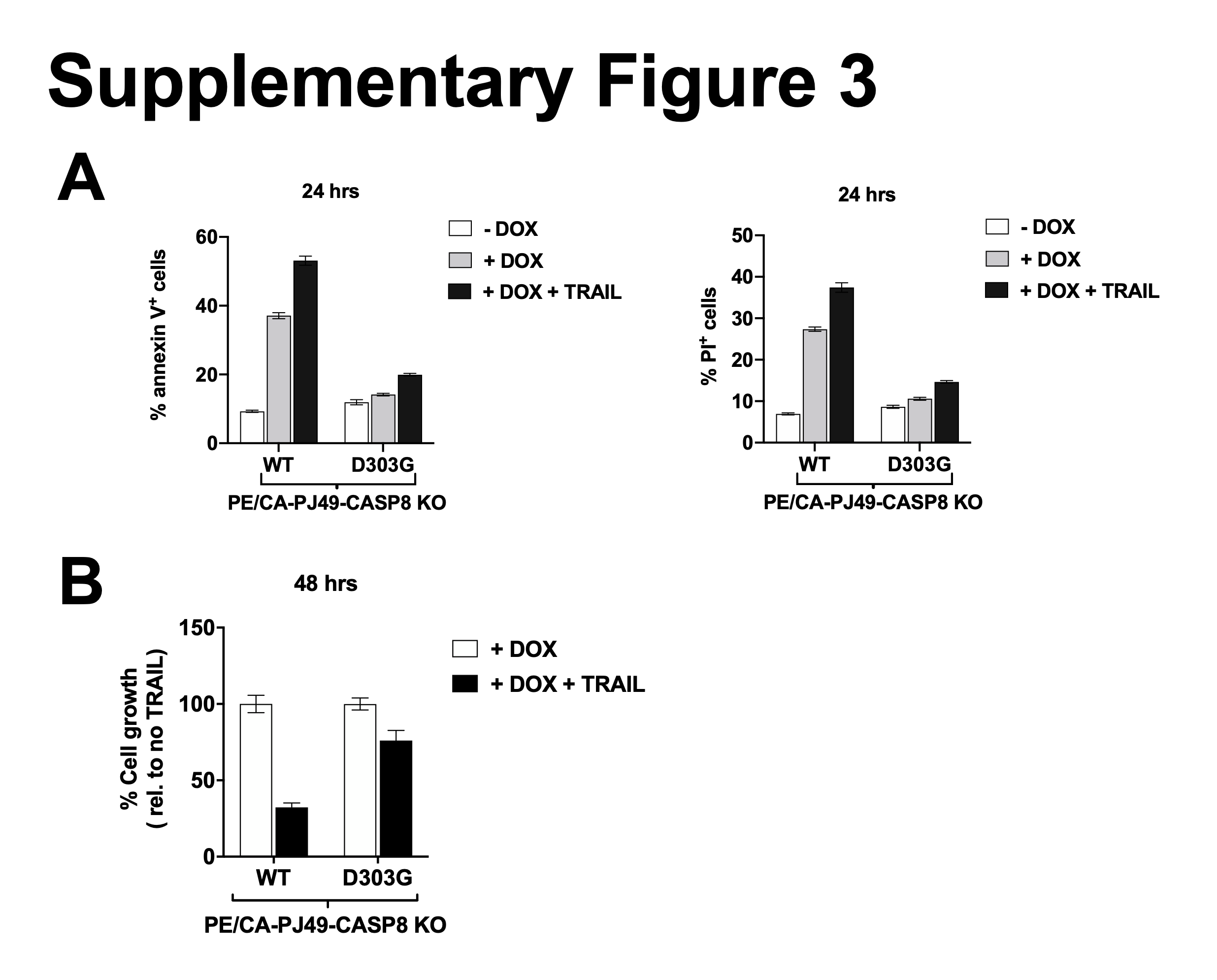

Supplement: Supplementary file 4 — Supplementary Figure 3A and 3B [file 41419_2021_4066_MOESM4_ESM.tif]

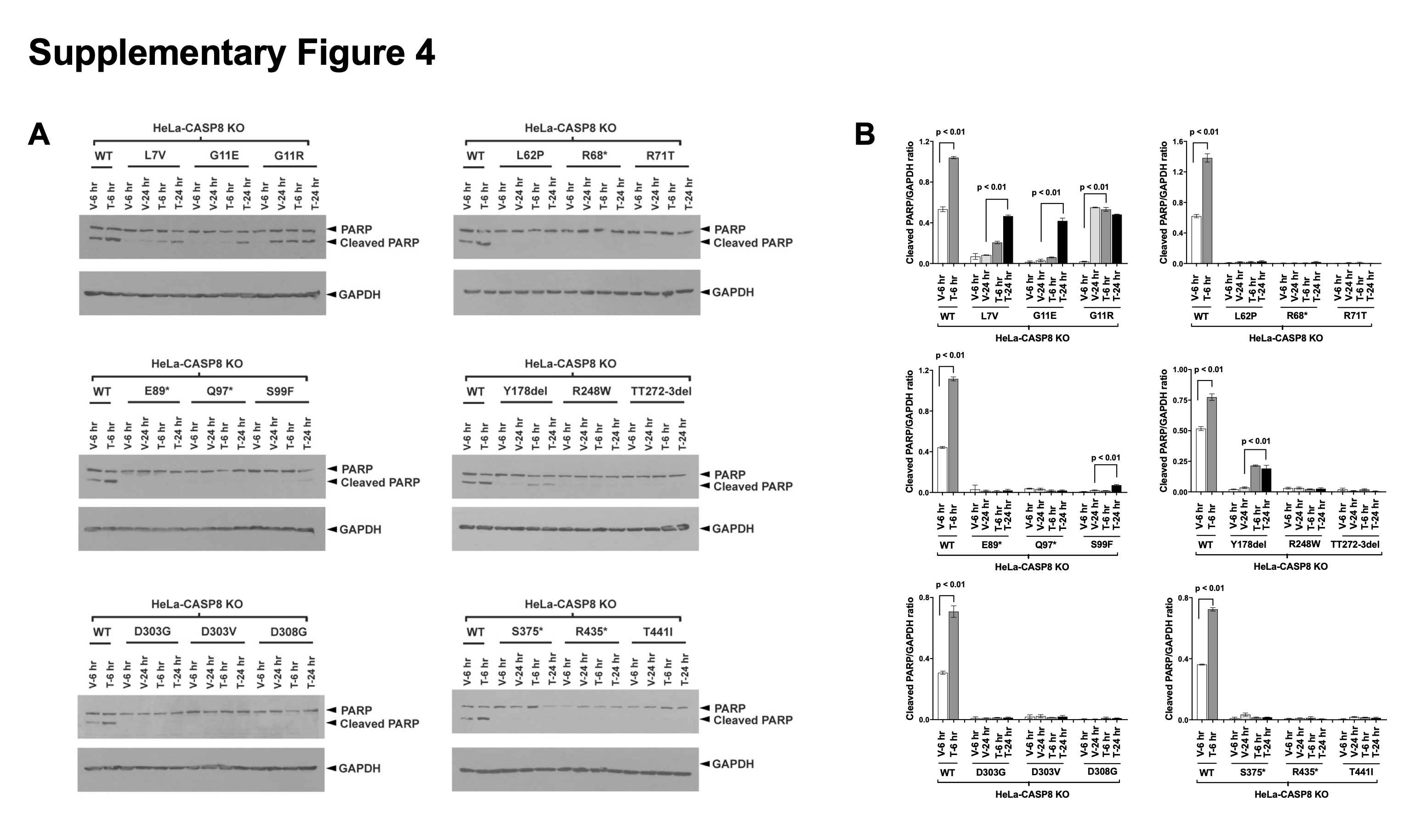

Supplement: Supplementary file 5 — Supplementary Figure 4A and 4B [file 41419_2021_4066_MOESM5_ESM.tif]

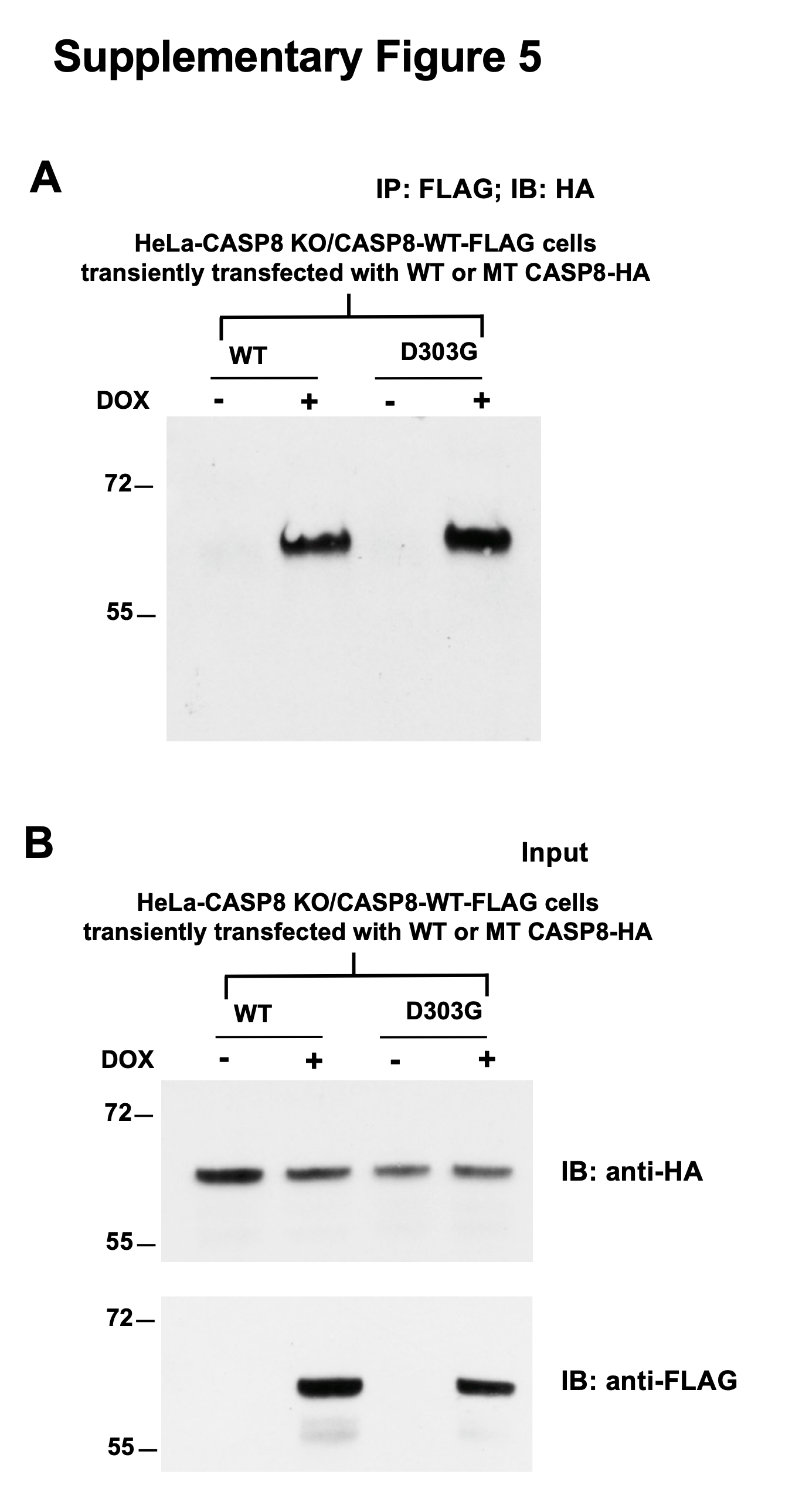

Supplement: Supplementary file 6 — Supplementary Figure 5A and 5B [file 41419_2021_4066_MOESM6_ESM.tif]

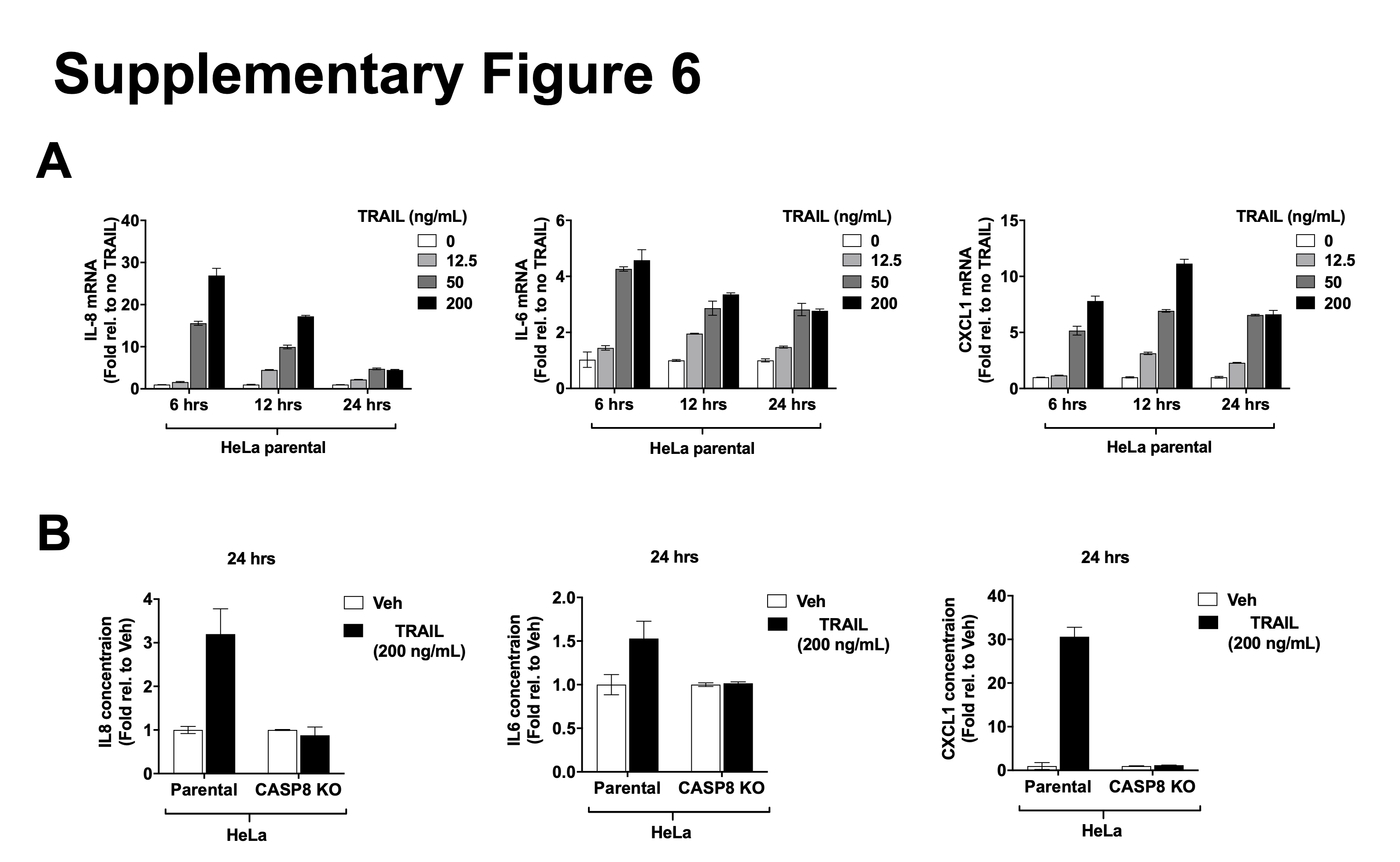

Supplement: Supplementary file 7 — Supplementary Figure 6A and 6B [file 41419_2021_4066_MOESM7_ESM.tif]

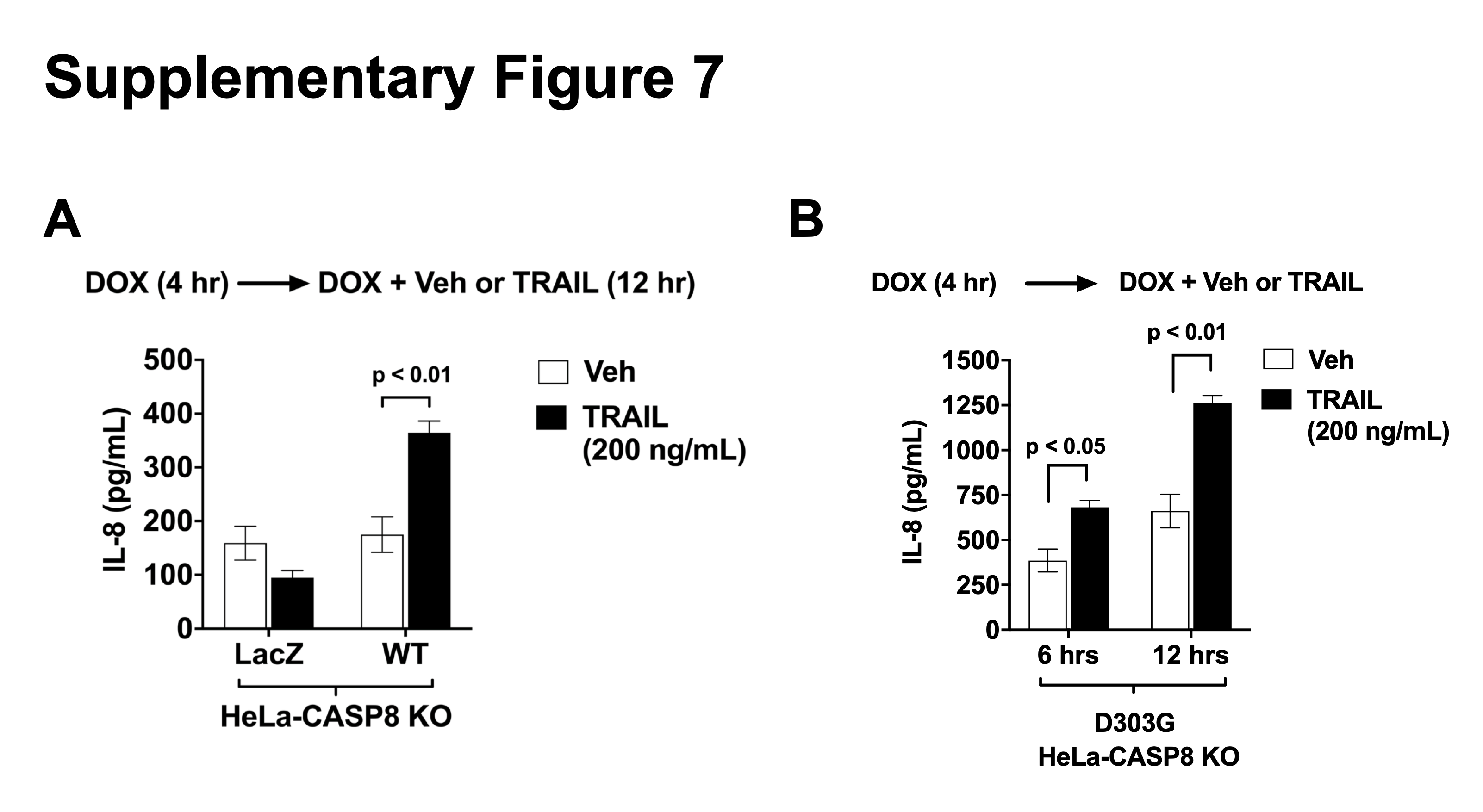

Supplement: Supplementary file 8 — Supplementary Figure 7A and 7B [file 41419_2021_4066_MOESM8_ESM.tif]

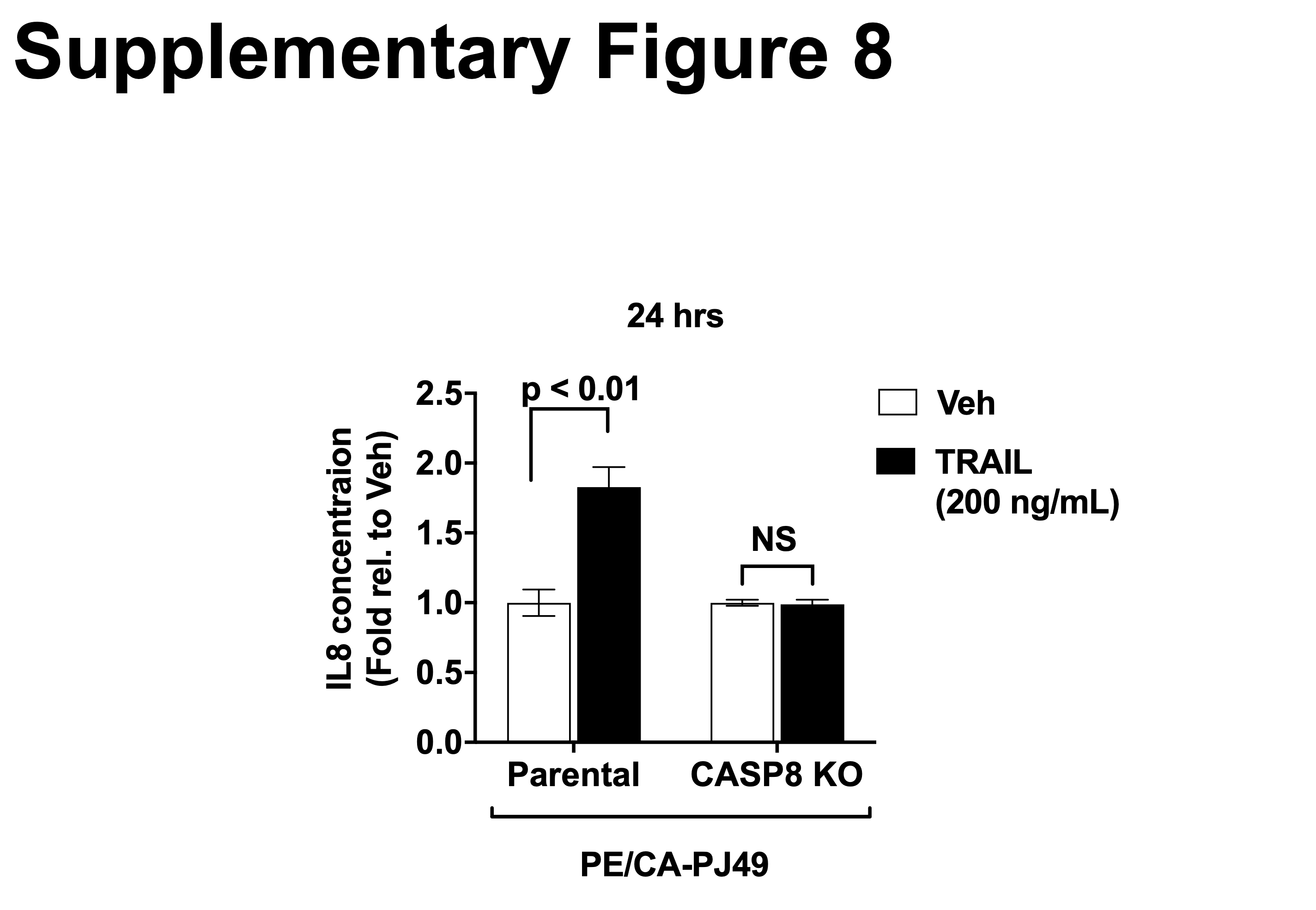

Supplement: Supplementary file 9 — Supplementary Figure 8 [file 41419_2021_4066_MOESM9_ESM.tif]

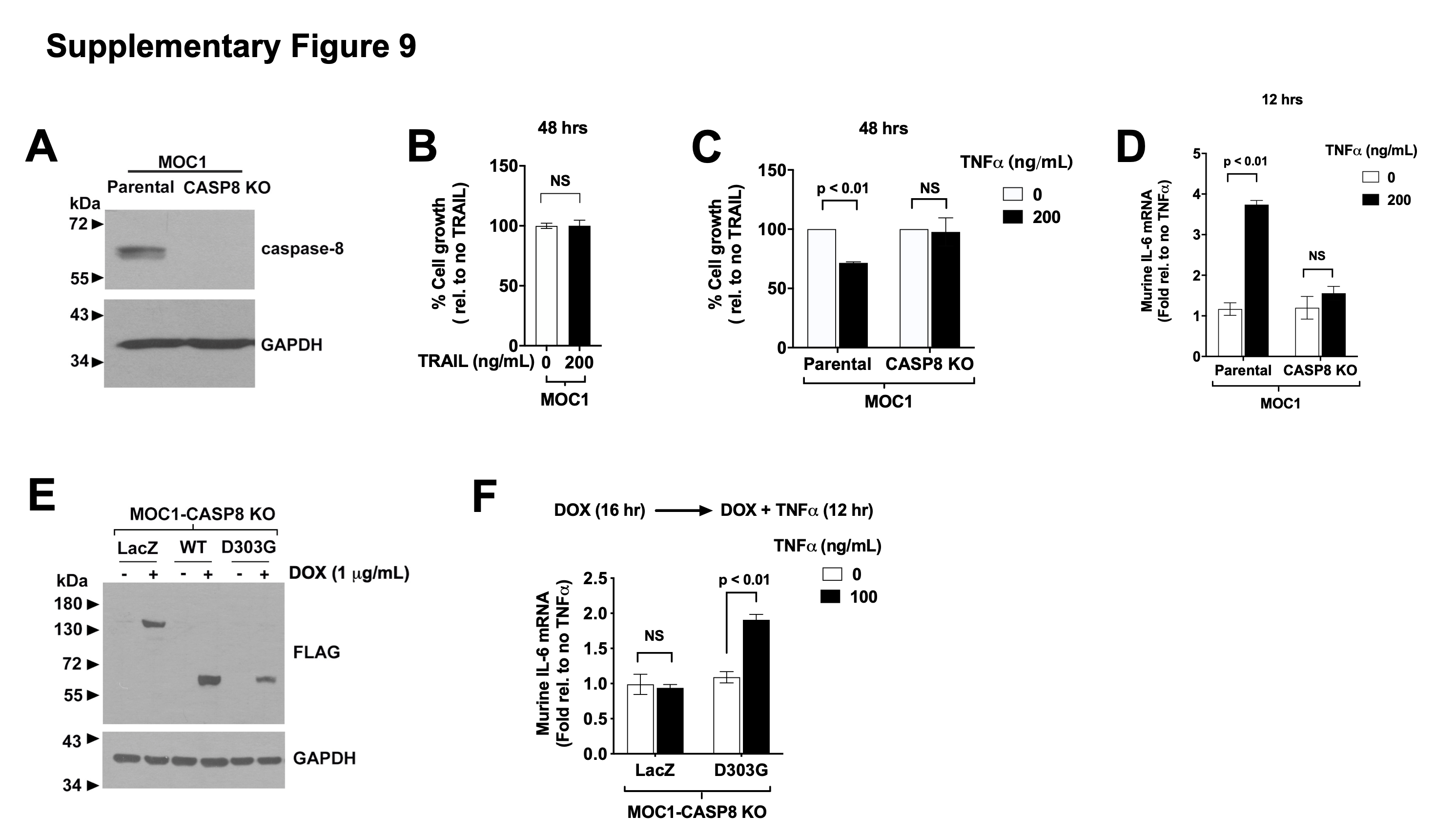

Supplement: Supplementary file 10 — Supplementary Figure 9A, 9B, 9C, 9D, 9E, and 9F [file 41419_2021_4066_MOESM10_ESM.tif]

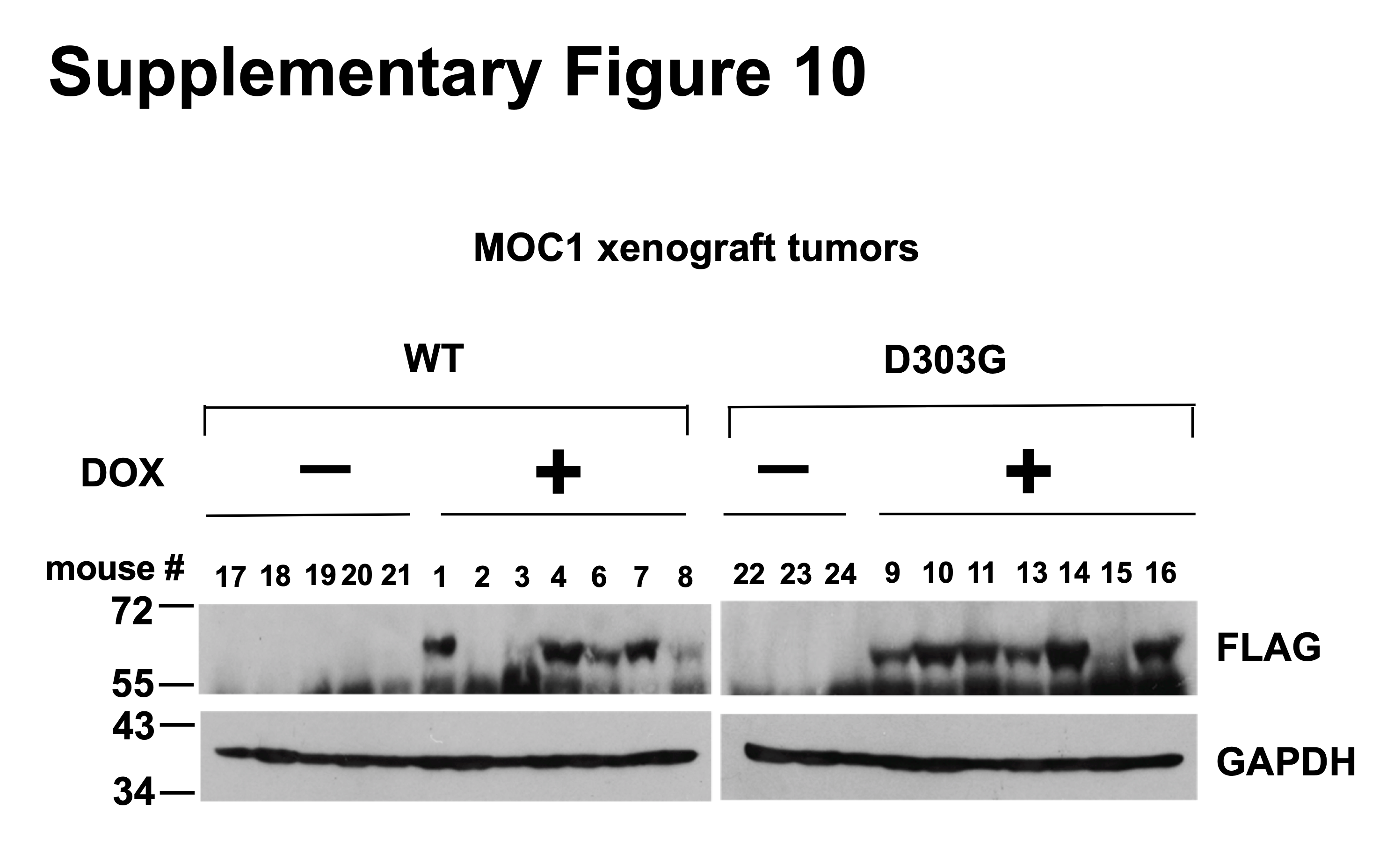

Supplement: Supplementary file 11 — Supplementary Figure 10 [file 41419_2021_4066_MOESM11_ESM.tif]

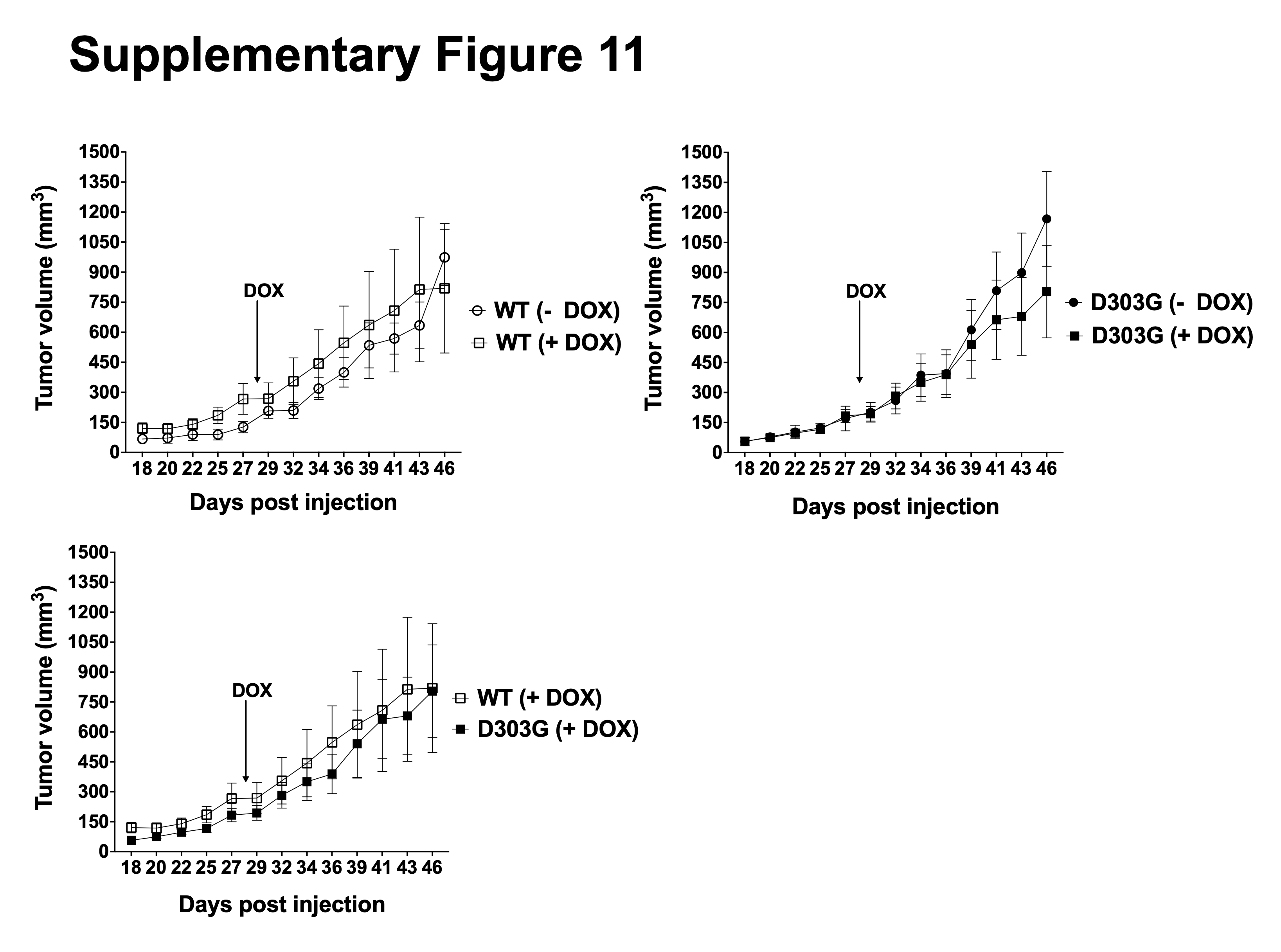

Supplement: Supplementary file 12 — Supplementary Figure 11 [file 41419_2021_4066_MOESM12_ESM.tif]
